# The Structural Sources of Verb Meanings Revisited: Large Language Models Display Syntactic Bootstrapping

**DOI:** 10.1162/OPMI.a.391

**Published:** 2026-10-03

**Authors:** Xiaomeng Zhu, R. Thomas McCoy, Robert Frank

**Affiliations:** Department of Linguistics, Yale University, New Haven, CT, USA; Wu Tsai Institute, Yale University, New Haven, CT, USA

**Keywords:** syntactic bootstrapping, verb learning, large language model, language learning, distributional hypothesis, lexical semantics, word learning

## Abstract

Syntactic bootstrapping (Gleitman, 1990) is the hypothesis that children use the syntactic environments in which a verb occurs to learn its meaning. Existing evidence for this hypothesis generally involves controlled experimental settings (e.g., Jin & Fisher, 2014; Naigles, 1990; Yuan et al., 2012). In this paper, we leverage the powerful statistical learning abilities of neural language models to examine the contribution of different kinds of cues for word learning when a learner is presented with a corpus of English linguistic input on the scale of a child’s experience. We do this by training two standard types of neural networks (masked and autoregressive transformers) on perturbed datasets where different sources of information relevant to word learning are individually removed. Our results show that models’ verb representations degrade more when we perturb word order, which removes syntactic information, than when we perturb co-occurrence among content words, which removes semantic information. Furthermore, the representations of mental verbs, for which syntactic bootstrapping has been shown to be particularly crucial in human verb learning, are more negatively impacted in the training regime with perturbed word order as compared to physical verbs. In contrast, while word order also affects models’ representations of nouns, the nouns are influenced more by co-occurrence information than the verbs are. In addition to reinforcing the important role of syntactic bootstrapping in verb learning, our results demonstrate the viability of testing developmental hypotheses on a large scale through manipulating the learning environments of large language models.

## INTRODUCTION

A fundamental question in language acquisition is how children acquire word meanings (Bloom, 2000; Carey, 1978; Chomsky, 1993; Gelman et al., 1998; Waxman & Lidz, 2006). An intuitive and long-standing answer is that they do so by observing word-world co-occurrences, learning word meanings by keeping track of the situations in which words occur and associating the common situational properties with the word meaning (Locke, 1847). For example, if the word *cat* most often occurs in scenes that involve a small domesticated feline animal, then children might make the inference that *cat* refers to this type of object. While this account might work for (concrete) nouns (Snedeker, 2000), the problem of verb learning poses a significant problem (Gleitman, 1990): the events in the world that correspond to verb meanings are often not observable at all. First, it is far from clear what specifically would constitute situational evidence for a seeing (as opposed to a looking) event. Additionally, a sentence like (1) involving the verb *see* might most naturally occur when its utterer is about to make a phone call and not actively looking at anything.

(1) Let’s see if Granny’s home!

Such failures in word-world alignment mean that cross-situational contingencies are an unreliable cue for verb meaning. These observations have led to an alternative view of how word meaning can be acquired: the proposal of **syntactic bootstrapping** (Gleitman, 1990; see also Brown, 1957 for an earlier version of this idea). This view argues that, in addition to word-world contingencies, the syntactic environments in which a verb occurs serve as an important source of information about its meaning. For instance, the fact that *see* can take a clausal complement, such as *if Granny’s home* in (1), provides a clue that *see* can be a mental verb that indicates something about the status of a proposition in a person’s mind.

In this paper, we examine the syntactic bootstrapping proposal through the lens of large language models (LLMs)—the types of neural networks, such as ChatGPT, that are central in state-of-the-art artificial intelligence systems. Current LLMs are built on the transformer architecture (Vaswani et al., 2017) and are trained on the objective of next-word prediction (Elman, 1990): given the start of a piece of text, they aim to predict the next word. This training typically proceeds over a very large corpus of text, currently as much as tens of trillions of words. LLMs replicate key aspects of human linguistic behavior, such as the time course of incremental processing (Wilcox et al., 2020, 2021), garden path effects (Futrell et al., 2019; van Schijndel & Linzen, 2018), and syntactic priming (Jumelet et al., 2024; Sinclair et al., 2022; Zhou et al., 2025), all in spite of the fact that syntactic structure is not explicitly encoded in the input or in the model. Although there are important differences between LLM and human learning (Frank, 2023) and behavior (e.g., McCoy et al., 2024; Weissweiler et al., 2025), there is nonetheless a growing body of work that has begun to explore the potential of LLMs to provide insight into the cognitive processes involved in language acquisition (e.g., Choshen et al., 2022; Evanson et al., 2023; Ficarra et al., 2025; Lavechin et al., 2022; Portelance et al., 2024). Because LLMs are powerful statistical learners that can learn effectively from naturalistic data, they enable controlled analyses of which aspects of a child’s input provide useful cues for the learning of different aspects of language. Previous studies have compared LLM and human acquisition of syntactic structure and lexical knowledge, often in isolation. While the recent work of Misra and Kim (2026) explores how lexical and structural cues jointly influence LLMs’ acquisition of novel verbs in dative constructions and has used these findings to generate novel developmental hypotheses, the interaction between syntactic and lexical knowledge remains unexplored beyond a construction-specific setting, particularly with respect to differences across lexical classes.

The current paper contributes to this line of work by using LLMs to explore the importance for lexical learning of the kind of structural information that syntactic bootstrapping proposes is important. Specifically, we examine the relative contribution of structural cues (operationalized as word order as LLMs operate over linear sequences of tokens rather than hierarchical structure) vs. contextual cues (operationalized as distributional co-occurrences: other words that co-occur with the verb) on the learned verb representations. While our primary focus is the syntactic bootstrapping hypothesis, the examination of contextual cues is also related to the lexical bootstrapping proposal (Goodman et al., 1998), which is the hypothesis that children use linguistic contexts to infer the meaning of novel words. To tease apart the respective contributions of structural and contextual cues, we selectively intervene on the training data in ways that remove information about either syntax or co-occurrence and test how these perturbations affect models’ learning of verbs. We find that verb learning is more negatively impacted by ablating syntactic information as compared to co-occurrence information, while nouns show a greater impact of ablating co-occurrence than verbs do (see Figure 1). In some cases, this asymmetry is manifested as nouns reversing the trend seen with verbs—resulting in a greater negative impact from co-occurrence perturbations than from word order perturbations. In other cases, this is manifested as nouns showing the same qualitative trend as verbs but a significant quantitative difference in the direction of nouns being more influenced by co-occurrence than verbs are. This asymmetry provides evidence that syntactic information does indeed play a significant role in the learning of verb meanings by LLMs, suggesting that structural cues are the more important source of information for verbs (as opposed to nouns, for which co-occurrences play a greater role than they do for verbs). The fact that LLMs display syntactic bootstrapping shows that naturalistic linguistic data contain syntactic cues useful in the learning of verb meaning, providing more naturalistic corroboration of conclusions previously drawn in controlled experimental settings. The presence of such cues means that humans and models can both capitalize on syntactic distributions as a common strategy for word learning during language acquisition, even in the absence of syntax-specific inductive bias.

**Figure 1.**
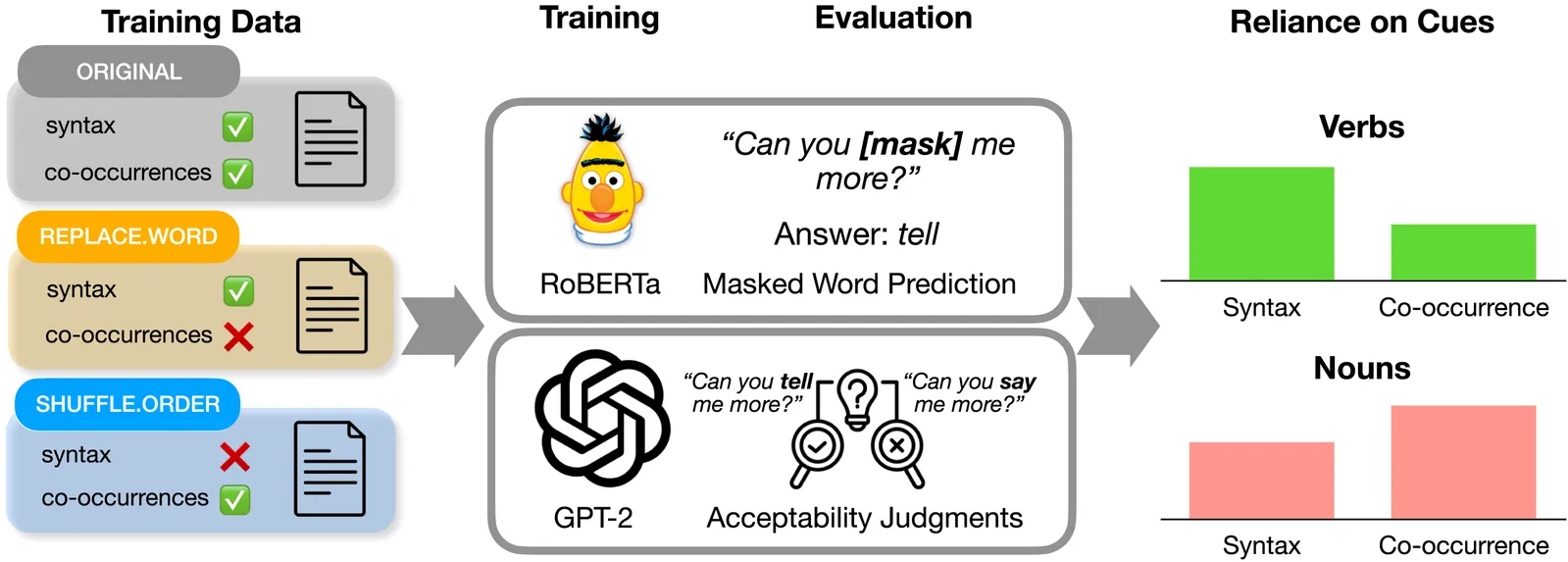
Our experimental setup involves three phases: creation of perturbed training data (left panel), model training (middle panel, left side), and evaluation of trained models on tasks that probe lexical representations (middle panel, right side). For the training data, replace.word removes co-occurrence information, while shuffle.order removes syntactic regularities, as compared to original, where both kinds of information are retained. We trained neural language models of two types of architectures: masked language models (RoBERTa; evaluated using masked word prediction) and autoregressive language models (GPT-2; evaluated using the minimal pair acceptability judgment task). The general characterization of the results is shown in the right panel. Verb learning relies more on structural cues, while nouns are more influenced by co-occurrence information than verbs are. Note that nouns and verbs sometimes show a qualitative reversal as shown here; in other cases, the nouns continue to show the same qualitative trend as verbs do, but with a quantitative difference that reflects a greater importance of co-occurrence in nouns than in verbs.

## BACKGROUND: EVIDENCE FOR SYNTACTIC BOOTSTRAPPING

While it is generally agreed upon that word-to-world mapping is a crucial information source for learning the first set of nouns (Gleitman & Trueswell, 2020), such a mapping is less informative for verbs. Many verbs are uttered in the absence of a corresponding event. Some verbs, such as those referring to mental states, do not refer to the observable world at all. And still others encode one of multiple perspectives that can be taken on a single event (e.g., *chase* vs. *flee*; *buy* vs. *sell*) and thus are impossible to identify uniquely on the basis of word-to-world mappings alone. These problems were the core of Gleitman’s motivations in proposing the syntactic bootstrapping hypothesis: children use the syntactic environments in which a verb occurs, together with its word-world co-occurrences, to infer its meaning. For example, upon hearing the sentence frame of (2a), a learner might infer that *verb1* encodes some caused action where Abigail is the causer and Betty is the undergoer.

(2) a. Abigail [verb1] Betty.
    b. Abigail and Betty [verb2].

In contrast, *verb2* in (2b) is more likely to encode an uncaused action, as Abigail and Betty are now both grammatical subjects who would both be undergoers. Under syntactic bootstrapping, a child could exploit this structural difference to focus on the kind of event that the verb in question describes.

A rich body of experimental work has provided support for syntactic bootstrapping (see Babineau et al. (2024) for a comprehensive review). For example, Naigles (1990) uses evidence from a preferential looking paradigm to argue that children around two years in age are able to use the grammatical differences between the examples in (2) to guide their interpretations: children associate novel verbs in transitive frames like (2a) with causative actions (such as an individual pushing another into a squatting position) and intransitive frames like (2b) with non-causative actions (such as an individual waving their arm). Such associations persist even in the absence of referential events during the first exposure to novel verbs (Yuan & Fisher, 2009). More recently, Perkins et al. (2025) examine the exact nature of such associations and find evidence that children employ the correspondence between syntactic structure and thematic relations (e.g., subject-agent, object-patient), instead of a more superficial proxy where the number of arguments in the sentence is mapped to the number of participant roles in the event (Yuan et al., 2012).

While such experimental work demonstrates that syntactic information can be used by children, it does not shed light on its relative importance as compared to other potential sources of information during the acquisition of verb meaning. To get at this question, Gillette et al. (1999) propose the **Human Simulation Paradigm** (HSP), where adult participants are asked to guess the common word that is produced by mothers across each of 6 muted videos showing them playing with their children. Results showed that the identification accuracy for nouns (45%) is significantly higher than for verbs (15%). More notably, one-third of the verbs are never correctly identified by any of the participants in the experiment, which underscores the difficulty of identifying verbs through cross-situational observation alone. Gillette et al. found that the identification accuracy for verbs is considerably higher when syntactic frame information (and no video) is provided to participants (51.7%) as compared to other conditions where cross-situational videos or a list of co-occurring nouns were provided. Furthermore, the verbs that are hard to identify from videos (namely mental verbs like *see*, *look*, and *want*) are the ones that are most easily identified when participants were provided with syntactic frames alone.

To sum up, the HSP successfully reveals what kinds of information are most useful in identifying word meanings as well as how nouns and verbs differ in this respect: **syntactic structure** is more helpful than **co-occurrence information** in predicting verb identities, while nouns can be much more easily identified via cross-situational observations. However, since the HSP only involves adult participants who are already fluent speakers of the language, it provides indirect evidence about how children use the different kinds of information present in their linguistic input during the process of word learning. In addition, the nature of experimental work imposes practical constraints on the length and variability of the experimental stimuli. As a result, such experiments cannot fully capture the scale and diversity of children’s linguistic input, including the wide range of contexts in which a word occurs and the potentially complex interactions between words and their contexts. In comparison, computational modeling can offer a complementary perspective into the learning problem by allowing systematic manipulation of input signals and observing learning performance over datasets on the scale of a child’s linguistic input.

## PROBLEMS OF ACQUISITION: INSIGHTS FROM LLMS

The advancement of neural language models has opened up new methods for examining puzzles of language acquisition, in a way that circumvents some of the aforementioned weaknesses inherent in human experimental work. There are few limits to the length and granularity of experimental manipulations we can conduct, and there are no ethical issues concerning the impact of such manipulations on the models’ attainment of language.

One notable kind of case where these models have led to progress concerns the question of innate domain-specific learning biases, such as the preference for hierarchical generalization (Chomsky, 1980; McCoy et al., 2020; Murty et al., 2023; Warstadt & Bowman, 2020; Yedetore et al., 2023). As weakly-biased artificial learners of human language, neural language models allow us to explore in great detail which aspects of natural language can be acquired in the absence of strong innate predispositions. If an aspect of language can be learned by a system that lacks strong innate predispositions, this provides evidence that at the very least such predispositions are not necessary to explain human language acquisition.

In this paper, we use the same type of computational model to investigate a different type of question: what is the role that different aspects of a learner’s linguistic input have in what the learner ultimately learns? Computational models are well-suited for such questions because they facilitate controlled manipulations of the data that learners encounter, something that is impractical and unethical to do with human participants. We focus on the problem of verb learning, specifically the syntactic bootstrapping hypothesis that was outlined in the previous section.

We aim to provide a computational parallel of the HSP, but adapted to a learning environment that encompasses the entirety of a child’s linguistic experience. We do this by removing different sources of information during learning and then comparing the linguistic knowledge gained by the learner in respective cases. This design is only possible in the case of artificial learners such as LLMs: the training data can be systematically manipulated to reveal the influence of specific linguistic constructions or properties during acquisition (Leong & Linzen, 2024; Misra & Mahowald, 2024; Patil et al., 2024). We therefore analyze the acquisition of English word meanings in LLMs to gain a window into which types of information in naturalistic linguistic input are most useful for lexical learning. Specifically, we explore the following research questions:

▪ RQ1: Do LLMs use syntactic information to learn verb meanings?
▪ RQ2: If so, do mental and physical verbs differ in terms of their reliance on syntax?
▪ RQ3: Does noun learning exhibit the same pattern as verb learning in LLMs?

To the best of our knowledge, no previous studies have examined these questions. Most of the previous literature on LLM meaning acquisition focused on the role of distributional information (Mikolov et al., 2013), lexical properties (Chang & Bergen, 2022), or nouns (Huebner & Willits, 2018, 2020, 2023) specifically, without examining the importance of syntactic information. While Sinha et al. (2021) did study the effect of word order in training on downstream natural language processing tasks (e.g., classifying whether a sentence has a positive or negative sentiment), they did not examine its effect on learning fine-grained aspects of linguistic meaning, such as verb meaning. This is the gap we aim to address. As a preview of our results, we found that syntactic information plays a more important role in verb meaning acquisition than co-occurrence information, where the reliance on syntax also differs between mental and physical verbs. In contrast, co-occurrence information is more crucial for noun learning as compared to verbs. Finally, we discuss the implications of our results with respect to syntactic bootstrapping and computational modeling of developmental hypotheses in general.

## METHODS

We train all language models from scratch based on three versions of the training set, which is in English: the original dataset, a perturbation that removes syntactic information, and a perturbation that removes co-occurrence information for verbs. We train LLMs in these three settings while holding constant the architectures and training algorithm. We then evaluate models on a targeted evaluation set for verb meaning. The respective roles of syntax vs. co-occurrences can then be assessed through performance differences.^1^

### Models

All models that we train are *transformers* (Vaswani et al., 2017), which are the most prevalent type of neural network for modeling language. Words in our dataset are first tokenized into subwords (tokens), each of which is encoded using a vector (for more details about the subword tokenization scheme, see below). These vector representations are then fed into stacked transformer layers. In each layer of the architecture, a new representation of the input tokens is computed through an *attention mechanism* which selectively synthesizes information from other tokens in the sequence. The synthesized representation is then passed onto the next layer. After the final layer, the embedded representations are projected back to the model’s vocabulary to generate a probability distribution over tokens.

We use two different types of transformer language models: *masked* and *autoregressive*. Both types have the same basic computational architecture, but they differ in the nature of the prediction task that the model is trained to perform. For masked language models (MLMs), some tokens are randomly masked in the input sentence during training, and the model is optimized to correctly predict the identity of the masked word using the surrounding context (Devlin et al., 2019). Autoregressive models, on the other hand, are optimized to predict the next token in a sequence of words based on the preceding context. We examine two types of transformer models to see if our results will generalize across different training objectives. Since masked language models make use of bi-directional information while autoregressive models are inherently uni-directional, it is possible that the relative contribution of syntactic vs. co-occurrence information differs across model architectures.

For the MLM, we used the architecture of BabyBERTa (Huebner et al., 2021), a model which has been used in studies of child language learning. BabyBERTa is a small version of RoBERTa (Liu et al., 2019), scaled down to include only 8 layers, 8 attention heads per layer, 256 hidden units, and an intermediate size of 1024 (14M parameters). For the autoregressive language model, we adopted the GPT-2 architecture (Radford et al., 2019)—specifically, the smallest size of GPT-2, which consists of 12 layers, 12 attention heads per layer, and 768 hidden units (117M parameters). This architecture has been adopted by a number of recent studies on developmentally-plausible training (e.g., Evanson et al., 2023; Misra & Mahowald, 2024), including work in the BabyLM challenge (https://babylm.github.io/; Hu et al., 2024; Warstadt et al., 2023).

### Data

The LLMs used in AI are typically trained on large-scale corpora scraped from Internet sources such as Wikipedia. To better simulate the experience of a child acquiring English, we use the developmentally-plausible corpus provided by the BabyLM challenge. We restrict our attention to the subset of this corpus that consists of child-directed speech from the English portion of the CHILDES corpus (MacWhinney, 2000). This yields approximately 3M sentences for our experiments, which comprise approximately 10M tokens.^2^

We divide this original data set into three subsets (or “splits”) which we use for training (85%), development (7.5%), and testing (7.5%). We also use original to create two perturbations that follow the same training, development, and testing splits: replace.word and shuffle.order. These are explained next, and examples are shown in Table 1.

**Table 1.**
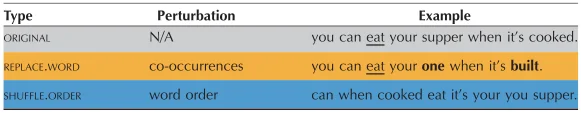
Summary of training perturbations and examples. Bolded words in replace.word are replacement words, while underlined words are the ones that remain unchanged from original.

#### replace.word.

In the replace.word perturbation, we remove the distributional co-occurrence information that is associated with a given verb. First, we use spaCy (Honnibal & Montani, 2017) to perform part-of-speech (PoS) tagging and dependency parsing. All other nouns, adjectives, adverbs, and verbs that co-occur with the root verb in the sentence are replaced with other words with the same PoS. Replacement words are sampled in proportion to their frequency within their respective PoS, which ensures that the overall frequency distribution is preserved. Controlling for overall frequency ensures that differences in model performance between training on replace.word vs. training on original can be attributed to the manipulation of co-occurrence information, rather than unintended changes in word frequency.

For sentences with multiple verbs, the main verb that is identified as the root of the dependency parse is left unchanged, while other verbs are replaced, with sampling done in the same way as other PoS categories. The resulting dataset retains the syntactic structure that is associated with a verb but eliminates potentially relevant co-occurrence information. Therefore, when we perform evaluations of verb learning, we expect a learner that depends mainly on syntactic information to learn verbs to do well when trained with replace.word, while a learner that relies more on distributional information will perform worse in the same situation.

#### shuffle.order.

In the shuffle.order perturbation, we remove syntactic dependencies signaled by word order that were present in original by randomly permuting the order of the words in the sentence. If models rely on syntactic information to learn verb meanings, training on shuffle.order will result in poor performance on an evaluation task for verb meanings. In contrast, if a model relies more on co-occurrence statistics (i.e., which words occur together with the verb), the model should succeed under this training regimen, as co-occurrence information of words in the same sentence is preserved. Sentences are shuffled on the word level rather than at the token level. We used the SentenceRandomizer algorithm proposed by Sinha et al. (2021), which has the advantage over completely random shuffling that no words remain in their original position (see Appendix C for pseudocode). This is particularly important given the nature of child-directed speech, where utterances are often short. Standard random shuffling could still result in some words occurring in their original position. By using the SentenceRandomizer algorithm, we ensure a more complete disruption of word order information across sentences of all lengths.

Critically, the amount of training data remains the same across all of our data sets, as neither perturbation changes the quantity of data. Thus, any differences seen across these conditions can be attributed to the type of data rather than the quantity.

### Training

Since our training data comes from child-directed speech, we trained a tokenizer on the original version of the data using the Byte-Pair Encoding algorithm (Gage, 1994; Sennrich et al., 2016), which incrementally merges together the most frequent pairs of adjacent symbols. We used the same tokenizer configuration for both model architectures, with the masked language model’s vocabulary additionally including the special <mask> token required by its training objective.

We trained the two architectures on each of the three dataset versions for 5 epochs, and each training configuration is repeated with 5 different random seeds. The training loss curves for each architecture, random seed, and dataset combination are included in the Appendix. Model checkpoint selection was based on validation loss from the original training dataset only. Specifically, among the five training epochs, we selected the checkpoint with the lowest validation loss in the original condition (i.e., epoch 5 for the masked language model and epoch 4 for the autoregressive model). To maintain a controlled comparison across perturbation conditions, we used the same epoch for all corresponding perturbed datasets (i.e., replace.word and shuffle.order). All the following results are averaged across these 5 random initializations. The training batch size and learning rate were determined via a hyperparameter search process. We found that for both architectures, models perform best under a learning rate of 1e−4. The optimal training batch size is 128 for RoBERTa and 256 for GPT-2.

### Evaluation

Because of differences in the models’ architectures, we adopted different evaluation tasks for probing how well models encode verb meanings. These tasks involve making judgments about a word’s appropriateness in particular contexts. This assumes that knowledge of a word’s distribution is a reasonable proxy for its meaning; see the Discussion for additional notes about this assumption.

#### Masked Verb Prediction.

To evaluate our models trained under the RoBERTa architecture, we took inspiration from the Human Simulation Paradigm (Gillette et al., 1999), where adult participants were asked to guess verbs. In our masked verb prediction (MVP) dataset, we mask out the verb in a sentence and evaluate the model on its ability to select the correct verb. Since we are interested in the effect of training perturbations on learned representations, the examples in this data set are selected from the original sequences in the BabyLM-CHILDES test set, holding the evaluation set constant for all models regardless of their training conditions. Simple sentence frames like *[mask] it!*, which occur frequently in child-directed speech, impose little constraint on the choice of verb. To make the evaluation more informative, we therefore filter our evaluation set only to sentences that are more than ten words long. In addition, we restrict our analyses to sequences in which the target verb is tokenized as a single token. The resulting test set contains 13,164 sentences with an average length of 12.94 words. For each sentence, we replaced the main verb with the mask token <mask> and then passed the modified sentence through the model. We then extract the probability distribution over the model’s vocabulary at the masked position. A prediction is considered correct if the top-ranked token corresponds to the original verb—e.g., if *play* is the top-ranked token when the model is given the masked version of (3):

(3) Original: How much does she play with stuffed animals at home?
    Masked: How much does she <mask> with stuffed animals at home?
    Correct: *play*

Since the vocabulary size of the tokenizer is 30,000, chance performance on MVP is $\frac{1}{30000}$.

#### Minimal Pair Judgment.

For models trained under the autoregressive architecture, the model cannot be evaluated on the task of MVP, as such models cannot make use of context following the masked token.^3^ Instead, we probe models’ knowledge of verb meanings by comparing the probability they assign to a sentence *S* from our test corpus with a sentence *S*′ that is derived from *S* through the replacement of the main verb by a different randomly chosen verb. The technical fact that enables such an analysis is that autoregressive models can be used to assign a probability to a sentence: such models are designed to assign a probability to each word given the sequence of words that came before it, and a probability for the whole sentence can then be produced by multiplying the probabilities for all of its words. Analogous to the evaluation of masked models, we also restrict our evaluation set only to sentences that are more than ten words long. We first identify the main verb in each example using spaCy (Honnibal & Montani, 2017). Second, we sample five other verbs within the same frequency bin as the original verb. This is achieved by ranking all verbs in the test corpus by descending frequency and binning them into four groups. This sampling method controls for the influence of frequency on the probabilities that models assign to sentences, as word frequency is known to strongly affect these probabilities (Lu et al., 2024; Tjuatja et al., 2025). This control makes probability differences within minimal pairs a more reliable measure of acceptability differences.

For each sentence, substituting the main verb with the replacement verb results in five alternative sentences, which correspond to five total minimal pairs. We use pyinflect, a Python module for word inflection that is an extension of spaCy, to ensure that the replacement verb has the same morphological inflection as the original verb. After replacement, we filter the sentence pairs to ensure that both sentences in each pair have the same number of tokens. The resulting test set contains 82,041 minimal pairs. Notice that our approach does not guarantee that all alternative sentences are ill-formed or even less acceptable than the original sentence. However, given the randomness of the replacement process, it is reasonable to assume that this will be the case in general. In addition, the use of multiple replacement verbs instead of one serves to minimize the effect that a specific replacement verb can have on the accuracy results.

(4) a. if you don’t **want** a new mother you better be good.
    b. # if you don’t **{play, push, give, stick, listen}** a new mother you better be good.

We evaluate each minimal pair test item 〈*S*, *S*′〉 separately, and we consider models to perform correctly if they assign a higher probability to the original sentence *S* than to the unacceptable sentence *S*′:

$$P\left(S\right)>P\left(S^{\prime }\right)$$ (1)

Therefore, chance performance is 50% for the minimal pair judgment task.

## EXPERIMENT 1: LLMS RELY MORE ON SYNTAX THAN CO-OCCURRENCE TO LEARN VERBS

The accuracy of masked and autoregressive LMs on the Masked Verb Prediction and Minimal Pair Judgment tasks is shown in Figure 2. In the original case, both architectures perform far above chance, indicating that our general setup is sufficient to enable models to learn a substantial amount about verb meaning. Turning to the perturbations, we find that both replace.word and shuffle.order have lower performance than original, suggesting that both syntactic information and word co-occurrences are helpful in learning verb meanings. More interestingly, across both architectures, models trained on the shuffle.order perturbation have the lowest accuracy, with the accuracy of replace.word being higher. The ranking original > replace.word > shuffle.order suggests that removing syntactic information has a larger impact on the model’s verb knowledge than removing co-occurrence information. We take this pattern to be evidence showing the important role of syntax for transformer language models to acquire verb meaning.

**Figure 2.**
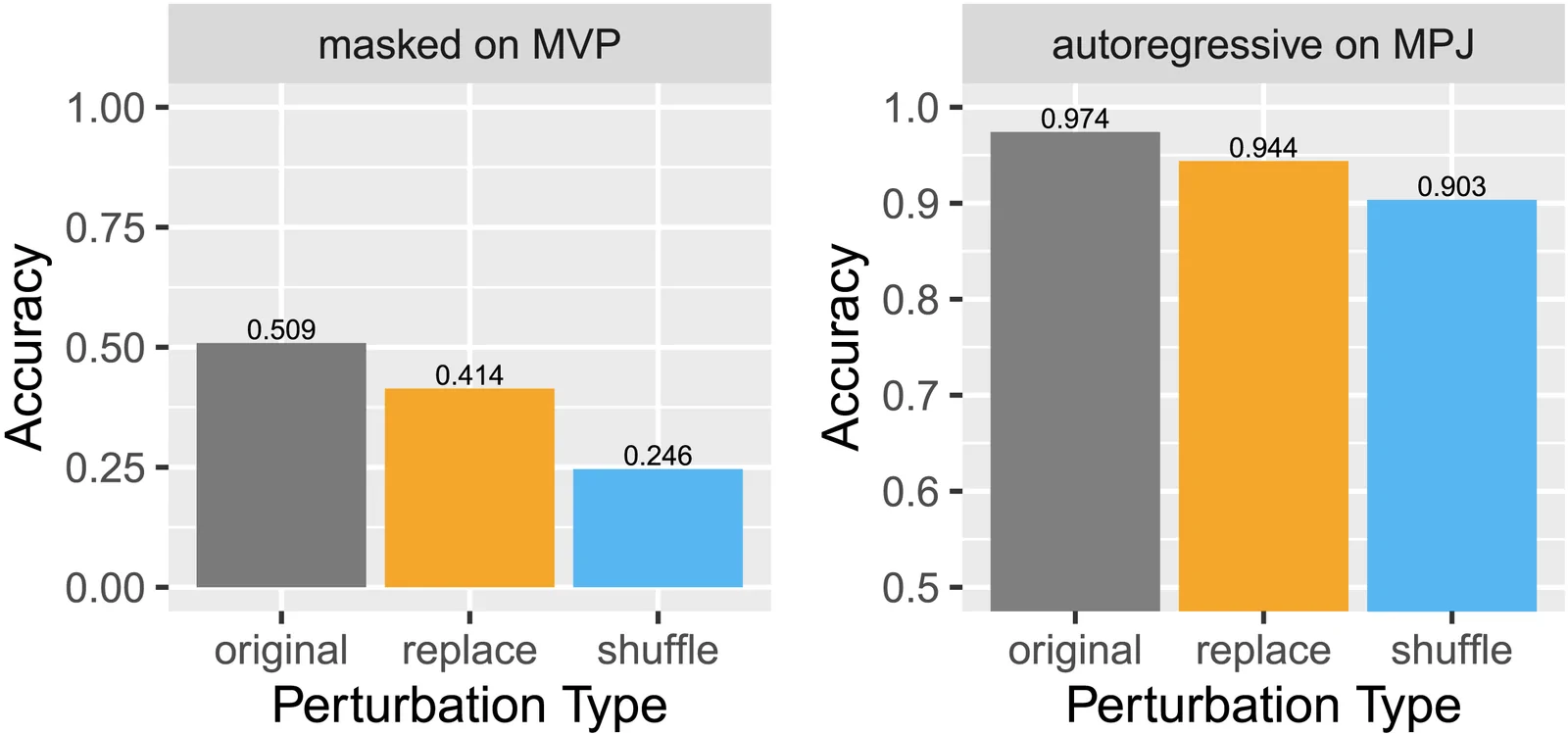
Accuracy of masked and autoregressive LMs on the Masked Verb Prediction (MVP) and Minimal Pair Judgment (MPJ) task, respectively, by perturbation type. Since each model is evaluated on a different task, chance performance is $\frac{1}{30000}$ for the masked model on MVP but 50% for the autoregressive model on MPJ. Note that the left vs. right panels have differing *y*-axes due to different performance ranges for the two types of tasks; the *y*-axis begins at chance performance level to emphasize differences above chance rather than absolute differences from zero.

### Mental vs. Physical Verbs

Recall from our review of the word learning literature that the identification of mental verbs in the Human Simulation Paradigm benefits more from syntactic cues than the identification of physical verbs does. Therefore, if transformer language models employ syntactic information in a similar way to human syntactic bootstrapping, we would not only expect to observe the original > replace.word > shuffle.order ranking but also an interaction between verb type and perturbation type. Specifically, the performance degradation from original to shuffle.order should be larger for mental verbs than for physical verbs. By our hypothesis, the degradation from original to replace.word should also give us a sense of how co-occurrence information affects verb learning. While we have no strong expectation a priori how mental vs. physical verbs might differ under the loss of co-occurrence, one possibility is that physical verbs might suffer more in this case, given their stronger reliance on context to determine aspects of their meaning.

By unpacking the results in Figure 2, we can examine whether this pattern holds. In particular, we restrict our analysis to test examples that involve only the mental and physical verbs studied by Gillette et al. (1999). The counts of each verb in the evaluation set are shown in Appendix A.

(5) a. Physical: *take*, *say*, *come*, *play*, *push*, *sit*, *pull*, *eat*, *make*, *call*, *catch*, *put*, *find*
    b. Mental: *see*, *look*, *want*, *know*, *like*, *think*

Results for MVP and MPJ evaluations with sentences involving only these verbs are shown in Figure 3. As expected, the original > replace.word > shuffle.order ranking continues to hold for both of these smaller sets of verbs across both architectures. To test for statistical significance, for each model architecture, we fit a logistic mixed-effects model using the lme4 package in R (Bates et al., 2015) to predict correctness on a test item using perturbation type and verb type (and their interactions) as predictors and example sentence as a random effect. Regression results are shown in Tables 2 and 3 for masked and autoregressive models, respectively.

**Figure 3.**
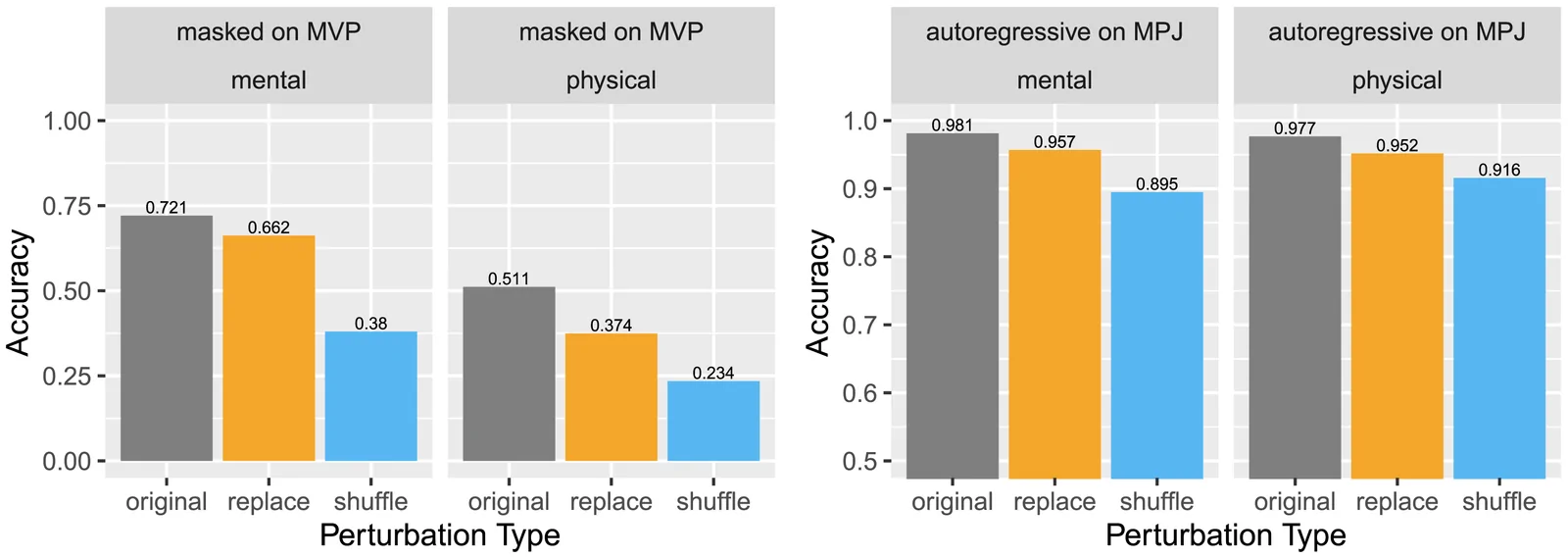
Accuracy on selected sets of mental or physical verbs by verb type, model architecture, and perturbation type.

**Table 2.** Logistic mixed-effects model predicting masked model accuracy with verb type and perturbation type.

**Parameter**	**Log-Odds**	***SE***	**95% CI**	***p***
(Intercept)	0.78	0.39	[0.02, 1.54]	0.043
perturbation [replace]	−0.30	0.02	[−0.35, −0.26]	<.001
perturbation [shuffle]	−1.58	0.02	[−1.62, −1.53]	<.001
verb type [physical]	−1.59	0.46	[−2.50, −0.69]	<.001
perturbation [replace] × verb type [physical]	−0.39	0.03	[−0.46, −0.32]	<.001
perturbation [shuffle] × verb type [physical]	0.07	0.04	[0.00, 0.14]	0.052

**Table 3.** Logistic mixed-effects model predicting autoregressive model accuracy with verb type and perturbation type.

**Parameter**	**Log-Odds**	***SE***	**95% CI**	***p***
(Intercept)	4.25	0.10	[4.06, 4.45]	<.001
perturbation [replace]	−0.88	0.03	[−0.94, −0.83]	<.001
perturbation [shuffle]	−1.87	0.02	[−1.92, −1.82]	<.001
verb type [physical]	−0.65	0.16	[−0.96, −0.33]	<.001
perturbation [replace] × verb type [physical]	0.11	0.04	[0.03, 0.19]	0.007
perturbation [shuffle] × verb type [physical]	0.48	0.04	[0.41, 0.56]	<.001

For both models, verb type is a significant main effect, which suggests that, in general, models have a higher accuracy in predicting mental verbs than physical verbs. Moreover, for both types of models, we find that the performance degradation from original to shuffle.order is bigger for mental verbs as compared to physical verbs, as we would expect if the acquisition of mental verbs relies more on syntax than physical verbs. For example, while the accuracy for physical verbs drops only 6 points from original to shuffle.order in the autoregressive LM, the corresponding degradation for mental verbs is 9 points. This is consistent with what the interaction terms suggest: the shuffle.order × verb type interaction is significantly positive for the autoregressive LM and marginally for the MLM, which means that the representation of mental verbs indeed suffers more than the representation of physical verbs when syntactic information is taken out from the training data.

On the other hand, the replace.word × verb type interaction term is negative and significant for the masked but instead positive for the autoregressive LM. This means that in the masked LM, the representation of physical verbs suffers more than the representation of mental verbs from losing co-occurrence information, while the same perturbation causes more disruption for mental verbs than physical verbs in the autoregressive model. However, it is worth noting that the magnitude of this interaction in the autoregressive model is small compared to the shuffle.order × physical interaction (0.48 vs. 0.11 in Table 3), indicating that differences between verb types are driven much more strongly by the removal of syntactic structure than by the loss of co-occurrence information. This difference in interaction is confirmed by a post-hoc contrast analysis using emmeans (*β* = −0.38, *SE* = 0.03, *p* < .0001) (Lenth & Piaskowski, 2026). In other words, disrupting word order has a substantially larger differential impact on mental versus physical verbs than disrupting lexical co-occurrence.

While it is not entirely clear to us why there might be a difference between the masked and autoregressive LMs in the replace.word × verb type interaction, it might stem from differences in parameter size and/or in the task that each architecture performs (i.e., masked language modeling vs. autoregressive next-word prediction). Differences in training objective might be particularly relevant here: masked language models have access to both preceding and following context during prediction, whereas autoregressive models only condition on preceding context. The following context may provide important cues to verb meaning, especially for mental verbs that are associated with particular complement types (e.g., CP rather than NP complements). This may make masked models less dependent on content-word co-occurrence for mental verbs than autoregressive models are. We leave the exact mechanism under which this might happen for future research.

Overall, the LLM results are highly consistent with the findings from the Human Simulation Paradigm in Gillette et al. (1999). Like humans, LLMs rely more on syntactic structure than co-occurrence information in learning verb meanings, and such dependence on syntax is stronger for mental verbs than for physical verbs.

### Impact of Shuffling Units

Within the current shuffle.order perturbation, sentences are shuffled on the scale of word unigrams (1gram). In this section, we consider an alternative shuffling condition np, where we first use spaCy (Honnibal & Montani, 2017) to identify base noun phrases and treat them as a single unit during shuffling (i.e., no NP-internal shuffling is performed). For example, as shown in (6), the words in the NP *your supper* retain their original linear order in np but not in 1gram.

(6) a. orig: you can eat your supper when it’s cooked
    b. 1gram: can when cooked eat it’s your you supper.
    c. np: when it [your supper]_NP_ ’s can cooked you eat.

1gram and np are most meaningfully distinguished in cases with more complex NPs, which might be relatively rarer in child-directed speech as compared to adult-directed speech. Nonetheless, the separation of these two subtypes allows us to examine the contribution to verb learning of local constituent structure, specifically that internal to NP, and the identity of the arguments to a verb, though not their syntactic relationship to the verb. If counting the number of argument NPs is more informative than counting the number of nouns (Yuan et al., 2012), we might expect models that are trained on np to have a better representation of verb meanings than those trained on the 1gram perturbation.

Results are presented in Figure 4, showing the performance for the two subtypes np vs. 1gram separately. np has a similar accuracy as 1gram for both the masked and autoregressive models, which means that preserving the internal structure of NPs is not sufficient as a source of syntactic information in verb learning. In other words, in order to successfully acquire verb meanings, it is important that sentence-level word order, which allows systematic verb-argument relations to be recovered, remain intact, as in original and replace.word.

**Figure 4.**
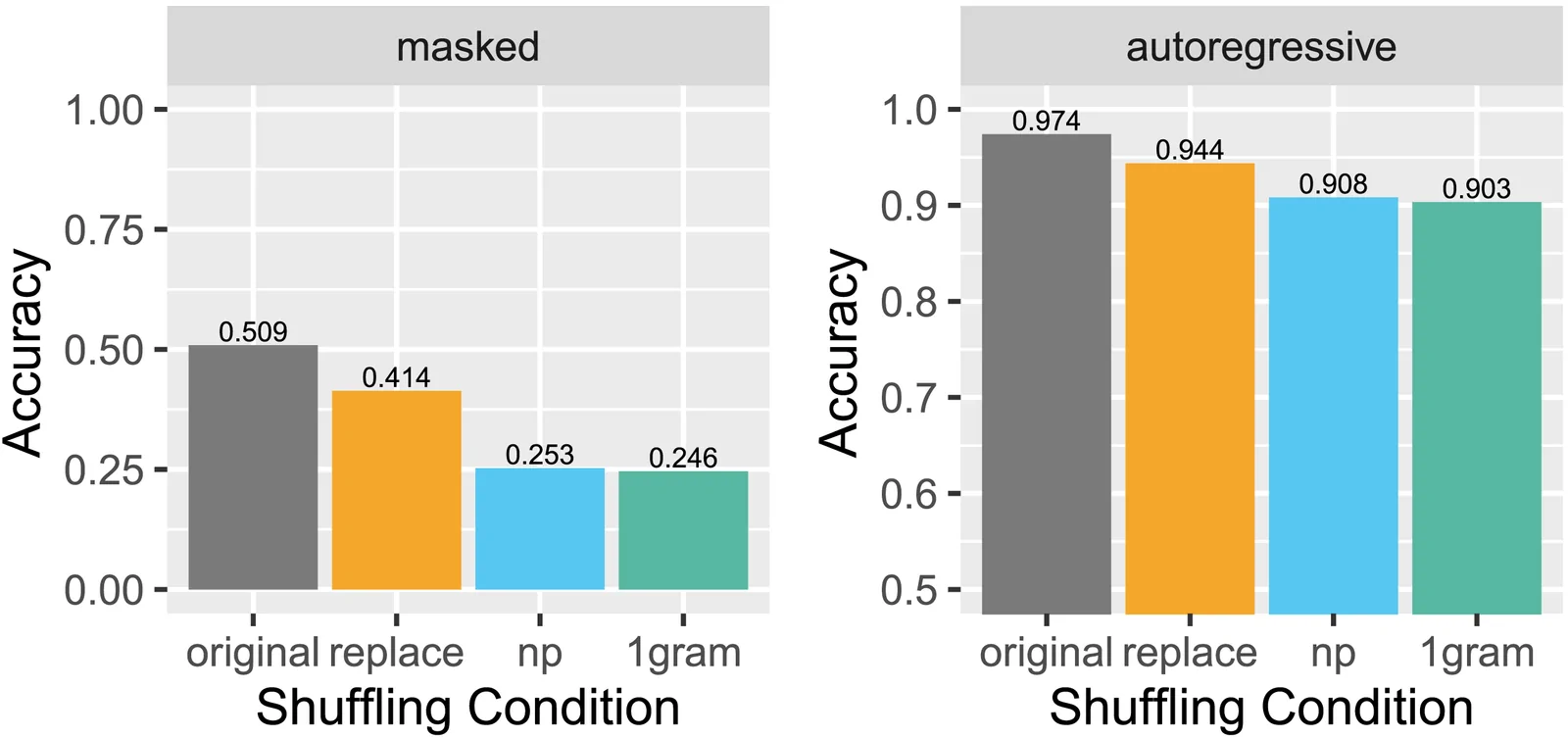
Reproduction of Figure 2 that includes an alternative shuffling condition np.

One line of existing work on child language acquisition proposes the structure-mapping account of syntactic bootstrapping (e.g., Fisher et al., 2020; Yuan et al., 2012), according to which children have an unlearned bias toward a one-to-one mapping between NPs in the sentence and participant roles in the corresponding event. In the initial stages of syntactic bootstrapping, children can make inferences about verb meanings not on the basis of the syntax itself but rather the simpler proxy of the number of NPs in the sentence (e.g., *He is gorping him* vs. *He is gorping*). Our results in Figure 4 suggest that having access to the kinds of patterns in the training data that allow for NP-counting is itself an insufficient general strategy for verb acquisition. Admittedly, our work does not directly speak to the validity of the structure-mapping account as an initial strategy during child acquisition, as we did not examine the performance of the model during early stages of training. However, our results do suggest that successful verb learning requires more syntactic information than the number of NPs in the sentence. Indeed, Perkins et al. (2025) found that children might instead adopt a content-based strategy where they use syntactic relations to infer thematic relations (e.g., subject-agent, object-patient). In English, the subject NP usually occurs before the object NP in a sentence, which is a systematic word order pattern that is removed in both np and 1gram subtypes of the shuffle.order perturbation. Future work could investigate these accounts from a computational perspective, examining how different types of syntactic cues might contribute differently across stages of verb acquisition.

## EXPERIMENT 2: LLMS RELY MORE ON CO-OCCURRENCE WHEN LEARNING NOUNS THAN WHEN LEARNING VERBS

The results of Experiment 1 demonstrate the important role of syntactic information during LLM verb learning. However, there is the possibility that the differential performance in the two different training conditions stems from the replace.word perturbation simply removing less information in some absolute sense than the shuffle.order perturbation. If this were the case, our results could be driven by differing amounts of information in the training data rather than a distinction between syntax and co-occurrence. We address this concern by considering the impact of the same manipulation on a different aspect of lexical learning, namely the learning of nouns. Results in the Human Simulation Paradigm suggest that, compared to verbs, the identification of nouns is much easier given situational cues. Moreover, Kako (2005) found that syntax was the least informative cue when adult participants were asked to guess the identity of nouns in the same paradigm.^4^ If this holds for LLMs as well, the learned representation of nouns when co-occurrence information is removed will be worse than that when syntactic information is removed. To test this prediction, we conduct a variant of our first experiment, focusing instead on nouns. All experimental setups are the same as Experiment 1 except for the replace.word training perturbation and the targeted evaluation set on nouns.

### Training Data

To examine the role of co-occurrence information on noun learning, we create a new replace.word perturbation. For each sentence, we randomly choose a single noun that occurs in it to hold consistent and replace all of the other open-class items (i.e., verbs, adjectives, adverbs, and nouns) with other words in the same part of speech. This perturbation is the exact parallel of the replace.word perturbation for verbs, where we replaced the nouns, adjectives, and adverbs that co-occur with a single verb. Examples under this perturbation are shown in Table 4 along with original and shuffle.order to demonstrate the difference.

**Table 4.**
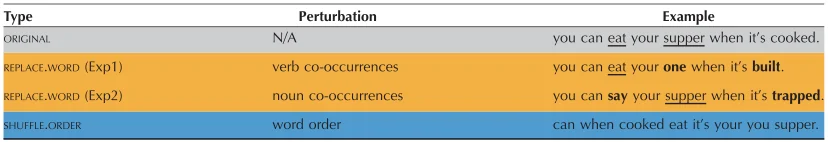
Reproduction of Table 1 with an additional row summarizing the data perturbation used in Experiment 2.

### Evaluation

The evaluation of noun representation closely follows that of verbs. In **Masked Noun Prediction**, the masked LMs were evaluated on whether they can correctly predict the masked noun in a sentence. The dataset contains 40,259 sentences with an average length of 14.02. In **Minimal Pair Judgment**, we compare a sentence with its counterpart where a single noun is replaced with a randomly chosen alternative noun, and the probability of each sentence was extracted from the autoregressive LM. Similar to the case of verbs, the replacement nouns were also sampled from the same frequency bin as the original noun. The resulting test set contains 95,231 minimal pairs. See below for examples:

(7) Masked Noun Prediction
    Original: but sometimes people also use it for breakfast in bed.
    Masked: but sometimes people also use it for <mask> in bed.
    Correct: *breakfast*

(8) Minimal Pair Judgment:
  - I’ll buy you one down the beach for ten cents.
  - # I’ll buy you one down the beach for ten **{movies, rolls, jars, ends, winters}**.

### Results

As shown in Figure 5, the accuracy pattern for nouns is qualitatively different from that of verbs. As we have seen for verbs (Figure 2), models under both architectures exhibit the ranking original > replace.word > shuffle.order. This ranking continues to hold for nouns under our masked model, but the difference between replace.word and shuffle.order becomes much smaller.

**Figure 5.**
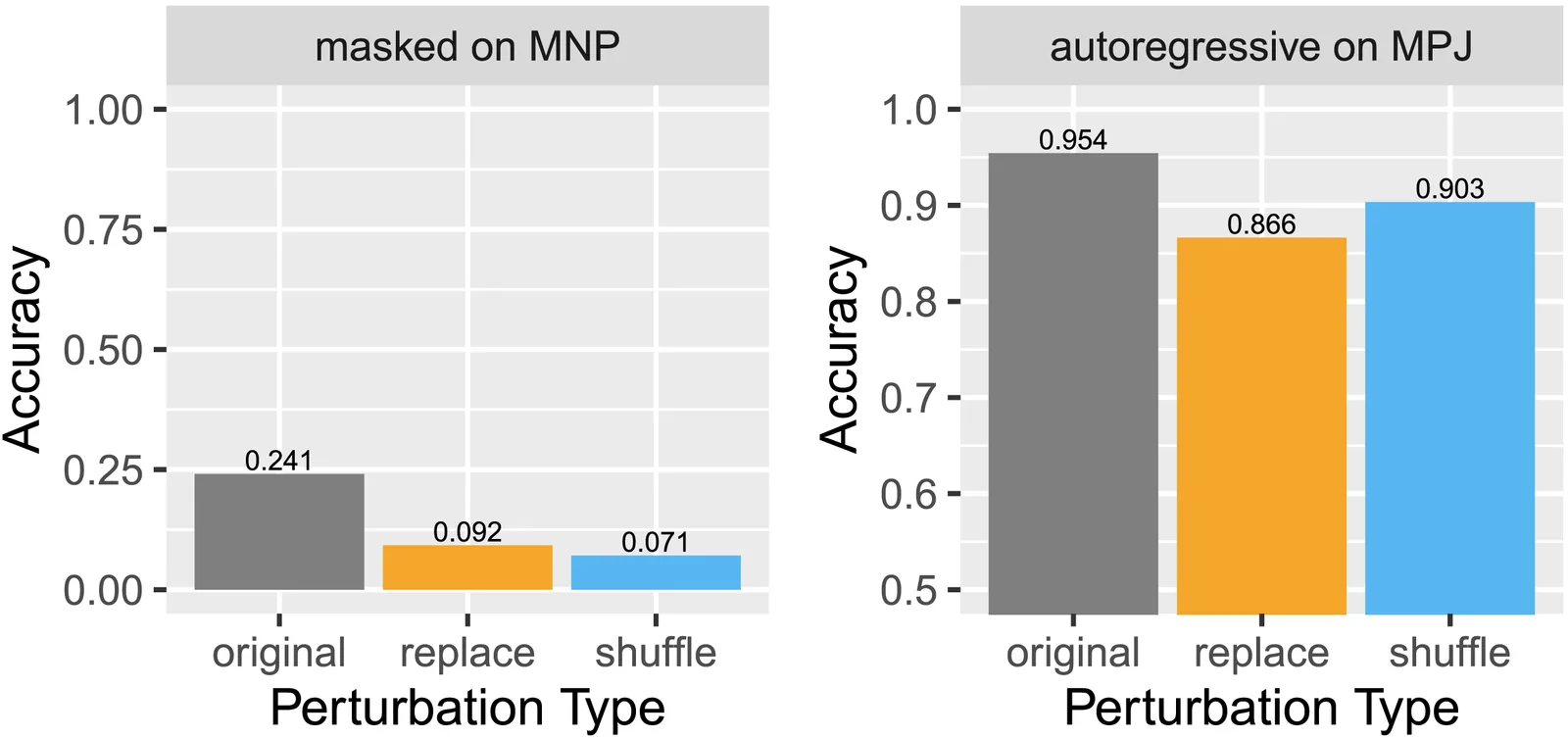
Accuracy on nouns by model architecture and perturbation type. Consistent with Experiment 1, masked LMs are evaluated under the Masked Noun Prediction task as in (7), while autoregressive LMs are evaluated under the Minimal Pair Judgment task as exemplified in (8).

To test for statistical significance, we fit a logistic mixed-effects model predicting masked models’ prediction performance with an interaction between part-of-speech and perturbation type, and with correct answer ID as the random intercept. Noun and original were selected as the reference levels. Regression results are shown in Table 5. There is a positive interaction between part of speech and replace.word (*β* = 0.96, *p* < 0.001), suggesting that noun learning relies more on co-occurrence information than verbs do. This finding is consistent with existing results in the human simulation paradigm. In addition, there is a positive interaction between part of speech and shuffle.order (*β* = 0.27, *p* < 0.001), which indicates that the reduction in prediction accuracy when syntactic information is removed is greater for nouns than for verbs. Overall, both positive interactions suggest that noun performance was affected more strongly than verbs in the masked models, regardless of the perturbation.

**Table 5.** Logistic mixed-effects model predicting masked model accuracy with part-of-speech (PoS) and perturbation type.

**Parameter**	**Log-Odds**	***SE***	**95% CI**	***p***
Intercept	−8.14	0.07	[−8.29, −8.00]	<.001
PoS [verb]	0.29	0.03	[0.22, 0.35]	<.001
perturbation [replace]	−1.54	0.01	[−1.56, −1.52]	<.001
perturbation [shuffle]	−1.89	0.01	[−1.91, −1.87]	<.001
PoS [verb] × perturbation [replace]	0.96	0.02	[0.93, 0.99]	<.001
PoS [verb] × perturbation [shuffle]	0.27	0.02	[0.23, 0.30]	<.001

On the other hand, when it comes to the representation of nouns under our autoregressive model, replace.word becomes the perturbation with the lowest accuracy: original > shuffle.order > replace.word. To test for statistical significance, we fit the same logistic mixed-effects model as previously for masked model performance. Results are shown in Table 6. There is a significant positive interaction for replace.word, indicating that the learning of verbs is less dependent on co-occurrence information than nouns. There is also a significant negative interaction for shuffle.order, which shows that the learning of verbs is more dependent on syntactic structure than nouns. The stark contrast between verb and noun performance further aligns with human results and emphasizes how the same training data perturbation can affect the learning of different linguistic elements in different ways.

**Table 6.** Logistic mixed-effects model predicting autoregressive model accuracy with part-of-speech (PoS) and perturbation type.

**Parameter**	**Log-Odds**	***SE***	**95% CI**	***p***
Intercept	3.22	0.03	[3.17, 3.28]	<.001
PoS [verb]	0.06	0.02	[0.02, 0.10]	.003
perturbation [replace]	−1.26	8.19 × 10^−3^	[−1.27, −1.24]	<.001
perturbation [shuffle]	−0.86	8.54 × 10^−3^	[−0.88, −0.84]	<.001
PoS [verb] × perturbation [replace]	0.43	0.01	[0.40, 0.45]	<.001
PoS [verb] × perturbation [shuffle]	−0.59	0.01	[−0.62, −0.56]	<.001

To sum up, the masked models exhibit different accuracy rankings for nouns than the autoregressive models: we observed original > replace.word > shuffle.order for masked models but original > shuffle.order > replace.word for autoregressive models. This difference is also reflected in specific interaction patterns in the corresponding statistical models. The interaction for replace.word is positive for both masked and autoregressive models. This is consistent with the hypothesis that, compared with verbs, there is larger reliance on co-occurrence information for predicting the identity of nouns. In contrast, the interaction for shuffle.order is negative for the autoregressive models but positive for the masked models. Therefore, the two model architectures differ in the direction of verb-noun asymmetry under the shuffle.order condition. Although our current methods do not allow us to pinpoint the exact reason why there is a difference between models, one possibility is that verbs and nouns draw on different structural cues, which might be affected differently by the two training objectives. For example, it is possible that predicting nouns relies more on the subsequent context than verbs. Since cues in the following context were distorted under shuffle.order, it could produce a larger decline in noun performance in masked models.

Nevertheless, results from the masked models are consistent with the broader pattern that the verb-noun difference varies across perturbation types. A post-hoc contrast analysis using emmeans shows that the verb-noun difference in the masked language model is significantly larger in the replace.word condition than in the shuffle.order condition (*β* = 0.69, *SE* = 0.02, *p* < .001). This indicates that the model performance on verbs was substantially more robust than nouns when co-occurrence information was removed, but only slightly more robust when word order was shuffled. Taken together, these results suggest that in masked models, verb learning is less dependent on lexical co-occurrence than noun learning, while both categories are sensitive to the loss of syntactic structure.

### Additional Evaluation Metrics

So far, the evaluation metrics we adopt for masked models are based on exact-match accuracy, where a prediction is counted as correct only when it matches the original target word. We chose this metric because it closely aligns with the Human Simulation Paradigm and thus allows for direct comparison with previous results. However, the exact-match accuracy fails to take into account the possibility that the sentence frames we provided the model with are compatible with multiple candidates. A similar issue arises for our MPJ task—since we use random sampling to generate alternative words, it is possible that both sentences in the pair are acceptable, although this possibility is partially reduced through averaging over multiple alternative verbs for the same frame. To examine whether our results depend on the exact-match metric for masked word prediction or on the sampling procedure for MPJ, we consider two additional metrics, one for each architecture, where predictions or judgments are validated by state-of-the-art LLMs.

For the masked models, we prompted GPT-5.4-mini (Singh et al., 2025) to evaluate whether the filled-in sentence is acceptable after inserting the masked model’s predicted word into the sentence frame. We refer to this metric as **LLM-based acceptability**, in contrast to exact-match accuracy. We report the proportion of predicted responses that receive a positive acceptability label. For the autoregressive models, we evaluate whether our model’s preference between the two sentences in each minimal pair is consistent with the acceptability judgments provided by Qwen3-8B (A. Yang et al., 2025). As an open-source model, Qwen3-8B allows us to directly obtain the probability it assigns to each sentence, which is not possible for GPT-5.4-mini. We refer to this metric as **LLM-based consistency** and report the proportion of pairs where the directions of acceptability judgments obtained from our model agree with those obtained from Qwen3-8B.

We refer readers to Appendix B for a complete set of results under the alternative metrics, including the mental vs. physical verb comparison in Experiment 1. Overall, results under the LLM-based metrics are highly consistent with the accuracy-based metric. There is a small caveat that for predicting nouns, replace.word and shuffle.order are quantitatively minimally different from each other under the accuracy-based metric. In comparison, under the LLM-based acceptability metric, the difference between replace.word and shuffle.order becomes more distinct (Figure B1; bottom left). However, the interaction patterns from the logistic mixed-effects models are consistent across metrics. Specifically, we find significant interactions between lexical category and perturbation type for the masked model: the verb × replace.word interaction is positive (*β* = 0.25, *p* < .001), as is the verb × shuffle.order interaction but with a much smaller magnitude (*β* = 0.09, *p* < .001). This is confirmed with a post-hoc contrast analysis using emmeans (*β* = 0.16, *SE* = 0.01, *p* < .0001). These results suggest that the effect of perturbation differs for nouns and verbs, which is consistent with an asymmetry between the two categories in their dependence on structural versus co-occurrence cues.

### Representation of Noun-Verb Distinction

The results we have presented above suggest that noun and verb learning exhibit qualitatively different sensitivity to structural vs. co-occurrence cues. This interpretation, however, assumes that the model maintains a meaningful distinction between noun and verb categories across all perturbations, including the shuffle.order condition where regular word order information is removed.

To verify this assumption, we applied principal component analysis (PCA) to the static embeddings of words that are unambiguously annotated as verbs or nouns and visualized their distribution in a 2D representational space. For the autoregressive model, we included 84 nouns and 84 verbs, each represented by a 768-dimensional embedding. For the masked model, we included 44 nouns and 44 verbs, each represented by a 256-dimensional embedding. We used equal numbers of nouns and verbs within each model to avoid class imbalance. See Figure 6 for the visualization across model architectures and perturbation types under one random seed. The results for the rest of the seeds are shown in Appendix E. Across all conditions, noun and verb representations remain visibly separable, suggesting that even the model trained under the shuffle.order perturbation encodes lexical category distinctions.

**Figure 6.**
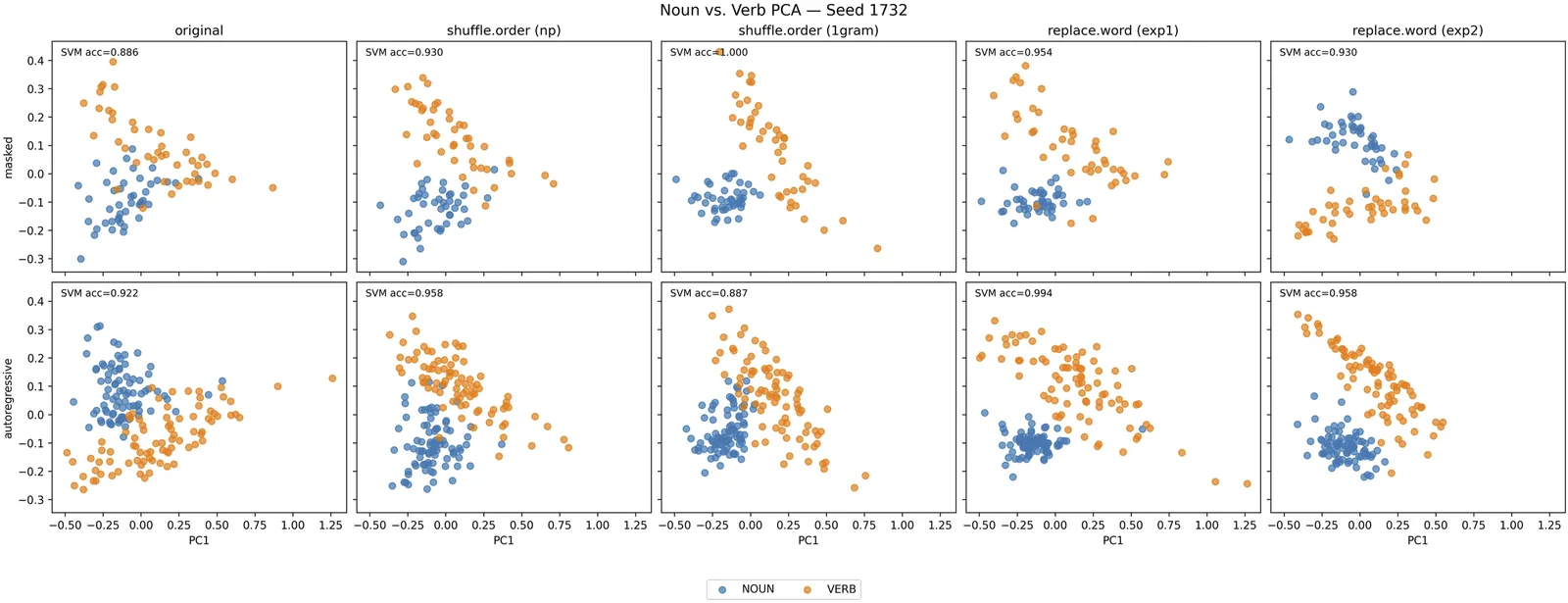
Visualization of noun vs. verb embeddings after PCA under the random seed 1732. Rows correspond to model architectures (masked vs. autoregressive) and columns to different perturbations.

To quantify separability between noun and verb embeddings, we trained a linear SVM classifier to distinguish noun and verb representations. Classification accuracy is shown in Figure 7. Across model architectures and perturbation conditions, classification accuracy remained consistently high (>90%). Crucially, high classification accuracy was also observed under both shuffle.order conditions. These results suggest that the behavioral differences we observed in previous experiments are unlikely to be explained by a collapse of noun-verb distinctions. They provide further support for our main finding: verb learning relies more on syntactic structure than co-occurrence information, while nouns rely more on co-occurrence information than verbs.

**Figure 7.**
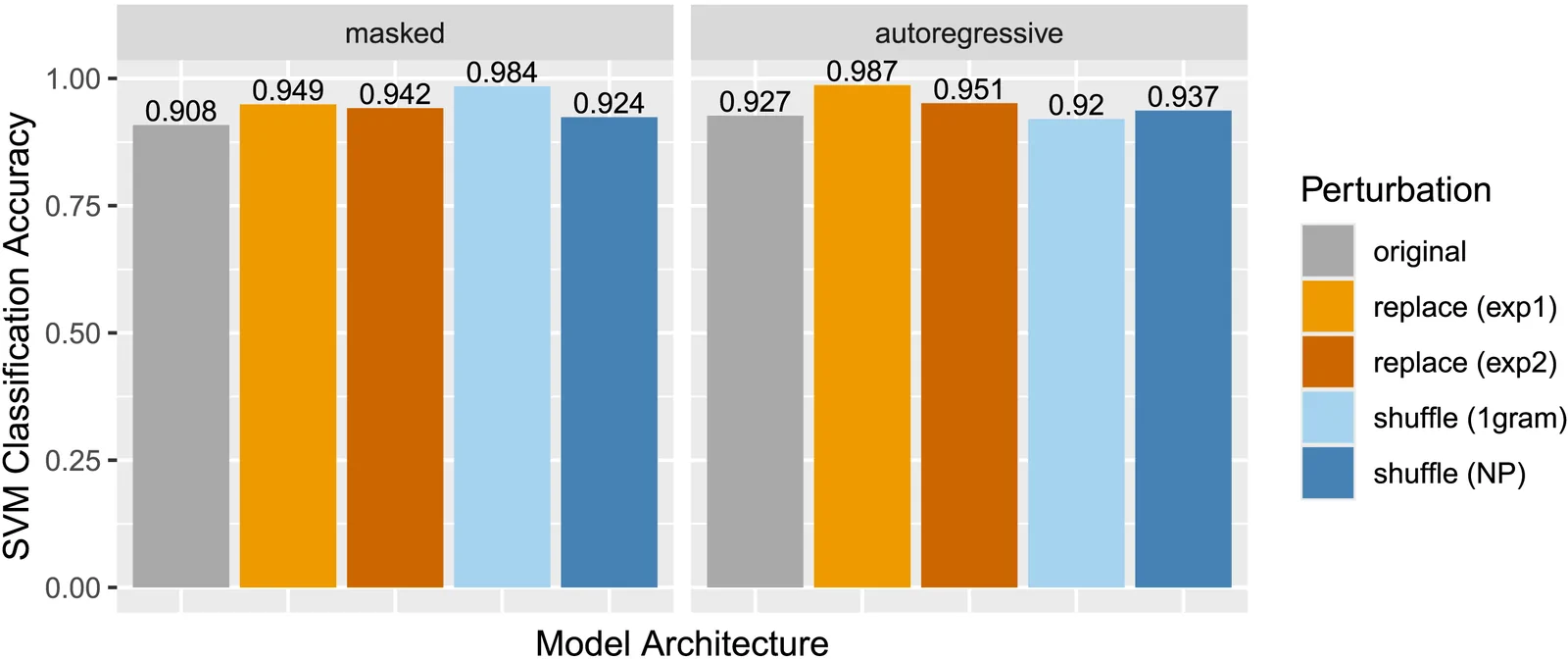
SVM classification accuracy by perturbation and model architecture averaged across random seeds.

## DISCUSSION

In this paper, we examined whether LLMs exhibit syntactic bootstrapping by training the masked and autoregressive LM architectures on perturbed variants of the CHILDES corpus: shuffle.order, which removes syntactic information, and replace.word, which removes the co-occurrence between verbs and the other content words that occur in the same sentence. Results showed a strong reliance on syntax for verb meaning: models’ accuracy on a verb meaning evaluation set decreases the most for shuffle.order, as compared to original. What’s more, both model architectures exhibit the same differing sensitivity to syntactic structure that humans do under the Human Simulation Paradigm (Gillette et al., 1999) in which mental verbs rely more on syntactic structure than physical ones. The consistency across both the masked and autoregressive LMs shows that the reliance on syntax for verbs is not an artefact of some specific model architecture or training objective, but rather is more likely to be a property of differing degrees of informativeness of the data that drives learning. This finding is strengthened in Experiment 2, where we showed that the acquisition of nouns exhibits stronger reliance on co-occurrence than verbs. Moreover, findings in both of our experiments align with results from the Human Simulation Paradigm, which examined the performance of adults rather than directly modeling the process of lexical acquisition. Despite the obvious differences in our experimental methods and participants (i.e., humans vs. LLMs), our results converge with previous experimental findings that verb meanings are more strongly correlated with syntactic structure while noun meanings are more closely tied to co-occurrence information.

### Distribution as a Proxy for Meaning

Our evaluation method in the first two experiments uses distribution as a proxy for meaning. We assume that models’ ability to accurately predict word identity (as in Masked Noun/Verb Prediction for masked LMs) or to (dis)prefer certain words (as in Minimal Pair Judgment for autoregressive LMs) in a given sentence frame is a reflection of their representation of verb meanings. This assumption follows if we adopt the Distributional Hypothesis (Firth, 1957; Harris, 1954), which holds that similarity in distribution corresponds to similarity in meaning. This hypothesis has been at the core of modern NLP developments such as word2vec embeddings, where the model is trained to predict a word using its neighboring contexts (Mikolov et al., 2013). The resulting word embeddings and their associated vector space successfully encode a wide range of semantic relations among words, including ones that are linguistically encoded (such as tense and gender) and those that involve encyclopedic knowledge. Our evaluation method for word learning focuses on the more behavioral side—we treat LLMs’ predictions and the probabilities that they assign to sentences as a reflection of their underlying encodings of meaning. While these predictions depend on the geometric properties of the learned word embeddings, we do not directly focus on the geometry but rather the behavior that results from the learned representations. In this respect, our design follows the setup of the Human Simulation Paradigm (Gillette et al., 1999), which draws conclusions about people’s ability to infer word meaning based on whether they can identify words from distributional cues, including syntactic frames and word co-occurrences. Given that prior work used similar setups, we believe that distribution is a reliable proxy for meaning and is a helpful way to assess meaning representations within LLMs.

### Operationalizing Contextual Cues

The syntactic bootstrapping hypothesis was proposed as an alternative to accounts suggesting that children acquire word meanings by observing word-world occurrences, where contextual cues correspond to extralinguistic information such as perceptual experience. In the current paper, we have operationalized contextual cues to word learning as word-word occurrence patterns, and disrupted these in the replace.word perturbation. On the one hand, our operationalization reflects the constraints of using text-only models, which do not have access to multimodal input. However, experiments using the Human Simulation Paradigm discussed above have already assimilated contextual cues with lexical co-occurrence: conditions in which experimental participants are given nouns co-occurring with a verb are as ineffective a contextual cue to meaning as non-linguistic, visual context in the form of silent videos. Nonetheless, a closer simulation of human language acquisition would involve, for example, training a vision-language model and perturbing the consistent correspondence between visual and linguistic information. However, as we discuss later, even incorporating such multimodality would still fail to capture the richness of the environment in which children learn language. As a result, our current setup should not be interpreted as a direct simulation of human acquisition, but rather as an investigation of how structural and distributional information differ in their support of lexical learning.

### Implications for Developmental Work

Overall, our results strengthen findings from the literature on the Human Simulation Paradigm and the syntactic bootstrapping hypothesis: even for a weakly-biased learner that does not have strong predispositions tailored to the structure of language, syntactic structure plays a greater role than co-occurrence information in determining learned verb representations. This means that the statistical regularities that exist between verbs and syntactic structure must be sufficiently stable in naturalistic data of the sort children experience during language learning, such that they can be usefully employed by any learner with an effective learning procedure that seeks to approximate the underlying distribution of language. Furthermore, the same types of information that facilitate human verb learning also support learning in machines.

To be clear, the current results provide evidence for an alignment between LLMs and humans in the types of information that they leverage for learning word meanings, but they do not speak directly to questions of whether LLMs and humans use the same learning mechanisms to exploit this information. Our findings highlight the important role of syntax during the LLM learning process: in order to arrive at an accurate representation of verb meaning and hence language, LLMs must somehow rely on syntactic structure. While the exact way in which such regularities factor into the learning process is beyond the scope of the current paper, our approach of transferring syntactic bootstrapping to LLMs identifies one direction that could, in principle, be unpacked on the level of computational mechanisms. Investigating these processes could shed light on the extent to which LLMs mirror human language acquisition and the plausibility of LLMs as cognitive models of the human mind.

### LLMs as a Tool for Testing Developmental Hypotheses on a Larger Scale

Our results also demonstrate how the paradigm of training data manipulation can facilitate theoretical and experimental work in language acquisition. Since our training data are taken from a developmentally plausible corpus, the results from models trained under different perturbations can reveal the kinds of cues that are available in the naturalistic input and their relevant contributions to the learning process. These manipulations are not possible with actual children, and child language experiments reveal behavior only in highly controlled and artificial settings. In comparison, our methodology not only allows for systematic selection of linguistic input but also simulates the entire course of acquisition, which extends the time scale on which the effects of a certain linguistic element can be observed. While the current paper only focuses on the endpoint of verb learning, future studies can generalize this methodology to other components of the acquisition process (e.g., adjective learning) and can consider the degree to which humans and LLMs differ.

### Parsing Child-Directed Speech

One potential limitation of our work is that our dataset generation process relies on an off-the-shelf spaCy parser to obtain part-of-speech tags and syntactic dependency relations. Given the child-directed nature of our dataset, we did observe parsing errors, particularly in sentences with ellipses. While such errors are unavoidable at the scale of our corpus, we expect them to introduce noise rather than contribute to systematic biases in our results. Recent resources would be likely to reduce such noise: UD-English-CHILDES (X. Yang et al., 2025) provides gold and silver dependency annotations for 48K sentences in CHILDES, and Zhou et al. (2026) addressed the difficulty in automatically and reliably identifying filler-gap dependencies in this domain. During the review process, CAIT (Padovani et al., 2026), a syntactic parsing toolkit specifically trained for child-adult interactions, also became available. These developments suggest that more precise structural annotations are possible, while also highlighting the challenges of scaling these techniques to the full corpus used in our experiments. Incorporating these improved parsing resources would allow for a more accurate manipulation of structural cues, and we leave this direction to future work.

### Trajectories of Word Learning

Although our work points to an alignment between humans and machines in verb acquisition, we did not examine the full learning trajectory. Instead, our analysis focused on representations after training had completed, leaving open the question of how such representations are acquired over the course of learning. As argued by Gillette et al. (1999), early verb learning requires some representation of syntax, which in turn requires the prior learning of a set of concrete nouns (which can be learned on the basis of contextual information, as we have seen). Future work could focus on examining how the use of these different information sources unfolds over time, building on our comparisons of verb and noun learning. Such an investigation would offer a fine-grained view of how learning in an LLM proceeds, opening up questions about the dynamic nature of the learning process that can be compared with human learning.

We have begun to explore this question by examining model checkpoints from training on the original dataset. Preliminary results suggest that directly comparing models’ performance on verbs vs. nouns is a non-trivial task—the two categories differ substantially in baseline difficulty, perhaps because the set of nouns is considerably larger than the set of verbs. It is therefore possible that the evaluation tasks we designed are inherently easier for verbs than nouns.^5^ In fact, our current data are consistent with this pattern: performance for nouns is consistently lower than that for verbs. It is possible that the observed differences reflect task difficulty rather than genuine differences in how models acquire verb and noun meanings. We believe this issue can be addressed in two ways: (i) by designing evaluation tasks that better balance the difficulty across lexical categories, and (ii) by adopting normalization methods that allow for a more controlled comparison between verb and noun performance. We leave these directions for future research.

### Extending to Other Languages

Our models are trained on child-directed speech only from English, which has a relatively strict word order. In English, word order encodes important syntactic information, although it certainly does not directly express all structural properties. In contrast, there are languages that have more flexible word order and/or rich morphological markings that encode syntactic relations in qualitatively different ways (e.g., Hungarian, Turkish). In these languages, the cues that encode syntactic structure, which thus could be used to support verb learning, might differ from those in English. The acquisition literature has examined whether the syntactic bootstrapping proposal generalizes to these typologically different languages. For example, Göksun et al. (2008) found that both the number of NPs and the accusative case marker affect children’s interpretation of verb meanings in Turkish. Because the specific syntactic cues that support verb learning may differ across languages, our current computational manipulation of word order may not disrupt structural information in other languages to the same extent as in English. Therefore, if our experimental setup were to be extended to other languages, different syntactic perturbations (e.g., manipulating case marking) may be required, and the current results may not generalize directly. We believe that cross-linguistic evaluation is an important direction for future work.

### Other Cues to Word Learning

The current paper only focused on the relative strength of two cues for verb and noun learning: syntactic structure (operationalized as word order) and co-occurrences of content words. Other syntactic and lexical-semantic signals might also play a role in this process, including cues based on function words, sentence length, specific syntactic frames, transitivity, subject–object order, and animacy. Since our current manipulations target the broad categories of word order and co-occurrence, the present results do not identify which of these more fine-grained cues drive the effects we observe. Isolating the strength of these components would help determine the most crucial factors for word learning, which we leave for future research. In addition, our work only examines linguistic cues to word learning, which in reality is a multimodal process grounded in social interactions (Bloom, 2000). While existing work has begun to explore whether neural networks can learn object names via visual grounding (Vong et al., 2024) and the benefit of multimodality for word learning (Zhuang et al., 2024), much remains unknown about how these information sources interact for all aspects of language. Extending our paradigm to other modalities could take us one step closer to modeling the actual environment in which children learn language.

## CONCLUSION

In this paper, we provide a new kind of support for syntactic bootstrapping, the claim that information about syntactic structure guides the learning of verb meaning. We leverage the capacity of LLMs to induce sophisticated representations of language from datasets at the scale of a child’s entire linguistic experience. Through interventions that are only ethical with computational simulations, we find that LLMs rely on syntactic structure to learn verb meanings in human-like ways: removing syntactic information causes larger degradation in verb representations than removing contextual co-occurrence. These results support the hypothesis that syntax plays a central role in word learning. In future work, the same methodology could be used to investigate the role of other cues in word learning.

## ACKNOWLEDGMENTS

We thank Athulya Aravind, members of the CLAY Lab, members of the Child Language Development Lab at Yale University, and audiences at the LSCP at the École Normale Supérieure, the Freeman Lecture at the University of Massachusetts, Amherst, and the Linguistics Department Colloquium at the University of California, Berkeley, for helpful feedback. We also thank the Wu Tsai Institute for providing access to the Misha computing cluster.

## AUTHOR CONTRIBUTIONS

X. Z.: Conceptualization; Formal analysis; Investigation; Methodology; Visualization; Writing – original draft; Writing – review & editing. R.T.M.: Conceptualization; Methodology; Supervision; Writing – review & editing. R.F.: Conceptualization; Methodology; Supervision; Writing – review & editing.

## DATA AVAILABILITY STATEMENT

All data and code are available at https://github.com/xiaomeng-zhu/structural-sources-verb-llm. The model checkpoints are available at https://huggingface.co/xmzhu/structural-sources-verb-llm.

## Notes

^1^ All model checkpoints, datasets, and results are available in our GitHub repository: https://github.com/xiaomeng-zhu/structural-sources-verb-llm.^2^ It has sometimes been suggested that training language models on child-directed speech facilitates the acquisition of syntax, though there are conflicting findings in the literature (Huebner et al., 2021; Padovani et al., 2025). While this question is interesting by itself, it is orthogonal to the purpose of the current paper, which focuses on the role of structural vs. distributional cues for word learning. One way of extending the current work would be to see if our results generalize to training on web-crawled corpora, though we leave this topic for future research.^3^ Masked language models can alternatively be evaluated using pseudo-log-likelihood scoring (Kauf & Ivanova, 2023; Salazar et al., 2020), which allows MLMs to be evaluated in a more similar way as autoregressive models. However, since the masked verb prediction task directly parallels the original human simulation paradigm, we retain it as our primary evaluation method.^4^ However, this is not to deny that syntactic cues were informative for acquiring nouns. In fact, it has been shown that children can leverage syntax to learn the distinction between word categories (e.g., de Carvalho et al., 2019) as well as granular differences within the noun class (e.g., Bloom & Kelemen, 1995).^5^ This distinction does not pose a problem for our current results, as we always compare across perturbations within each lexical category.

## Supplementary Material


