# Supplementary material for: The Structural Sources of Verb Meanings Revisited: Large Language Models Display Syntactic Bootstrapping
Source: Open Mind (Camb). 2026 Oct 3;10:1609–35. doi: 10.1162/OPMI.a.391 (PMC13733554; doi:10.1162/OPMI.a.391)
Supplement: Supplementary file 1 [file opmi-10-1609-s001.pdf]

A: DATASET STATISTICS

Table A.1 reports the number of sentences in the evaluation sets for both architectures for each mental verb. Table A.2 reports those for physical verbs.

Verb	Masked	Autoregressive
know	571	2855
like	359	1771
look	450	2255
see	545	2730
think	1689	8460
want	436	2155
**Total**	**4050**	**20226**

Table A.1. Distribution of mental verbs in the evaluation set of both model architectures.

Verb	Masked	Autoregressive
call	46	226
catch	0	40
come	323	1620
eat	246	1212
find	137	666
make	236	1157
play	189	929
pull	20	100
push	29	143
put	671	3355
say	596	2930
sit	139	686
take	280	1382
**Total**	**2912**	**14446**

Table A.2. Distribution of physical verbs in the evaluation set of both model architectures.

B: ALTERNATIVE METRICS OF EVALUATION

As discussed in Experiment 2, we consider two alternative metrics, one for each model architecture, respectively: an LLM-based acceptability metric for masked models and an LLM-based consistency metric for autoregressive models.

Results

Verb vs. Noun Results for the two lexical categories under the alternative metric are shown in Figure B.1. Overall, compared to the exact-match accuracy on MVP, models' performance under the LLM-based acceptability metric is substantially higher. This

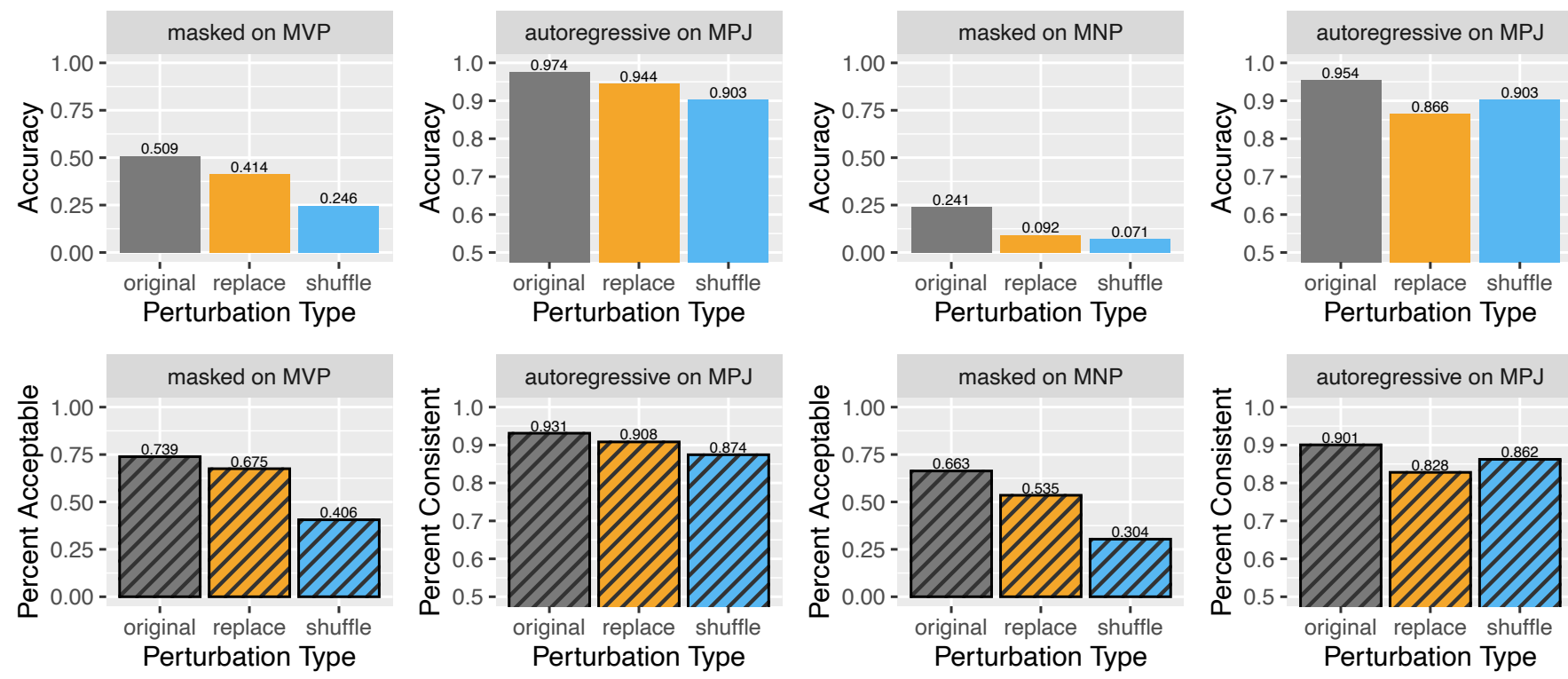

Figure B.1. Results under the accuracy metric (top row; replicating Figures 2 and 5) vs. alternative metrics by lexical category, model architecture, and perturbation type (color).

increase suggests that across all perturbations, there are some model predictions that constitute a reasonable response but are excluded by the exact-match criterion.

For verbs, the qualitative trend among the perturbation types (i.e., ORIGINAL > REPLACE.WORD > SHUFFLE.ORDER) remains unchanged under the new metrics.

For nouns, the autoregressive models exhibit an identical trend in the LLM-based consistency metric as in the accuracy-based metric (bottom-right): REPLACE.WORD is the perturbation with the lowest consistency judgments from `Qwen3-8B`, while ORIGINAL is the highest. For masked models (bottom-left), the accuracy-based metric and the acceptability-based metric yield the same trend (i.e., ORIGINAL > REPLACE.WORD > SHUFFLE.ORDER). However, under the accuracy-based metric, the quantitative difference between REPLACE.WORD and SHUFFLE.ORDER perturbations is minimal (Figure 5). This is not the case under the LLM-based acceptability metric — there is a 23% difference between REPLACE.WORD and SHUFFLE.ORDER.

To investigate the interaction between lexical category and perturbation type, we ran the same logistic mixed-effects model structure as before, but instead predicting acceptability/consistency. Despite the quantitative differences described above, it is important to note that the noun-verb distinction that we observed under the accuracy-based metric still holds for the LLM-based acceptability metric: the positive interaction for REPLACE.WORD was still larger in magnitude than that for SHUFFLE.ORDER (Table B.1). This pattern suggests that the asymmetry between lexical classes we observed is irrespective of the exact evaluation metric we adopt.

**Parameter**	**Log-Odds**	**SE**	**95% CI**	**p**
(Intercept)	0.42	0.02	[0.38, 0.46]	$< .001$
PoS [verb]	0.24	0.02	[0.20, 0.28]	$< .001$
perturbation [replace]	-0.57	0.007	[-0.59, -0.56]	$< .001$
perturbation [shuffle]	-1.60	0.007	[-1.61, -1.58]	$< .001$
PoS [verb] × perturbation [replace]	0.25	0.01	[0.22, 0.27]	$< .001$
PoS [verb] × perturbation [shuffle]	0.09	0.01	[0.06, 0.12]	$< .001$

Table B.1. Logistic mixed-effect model predicting masked model acceptability with lexical category and perturbation type.

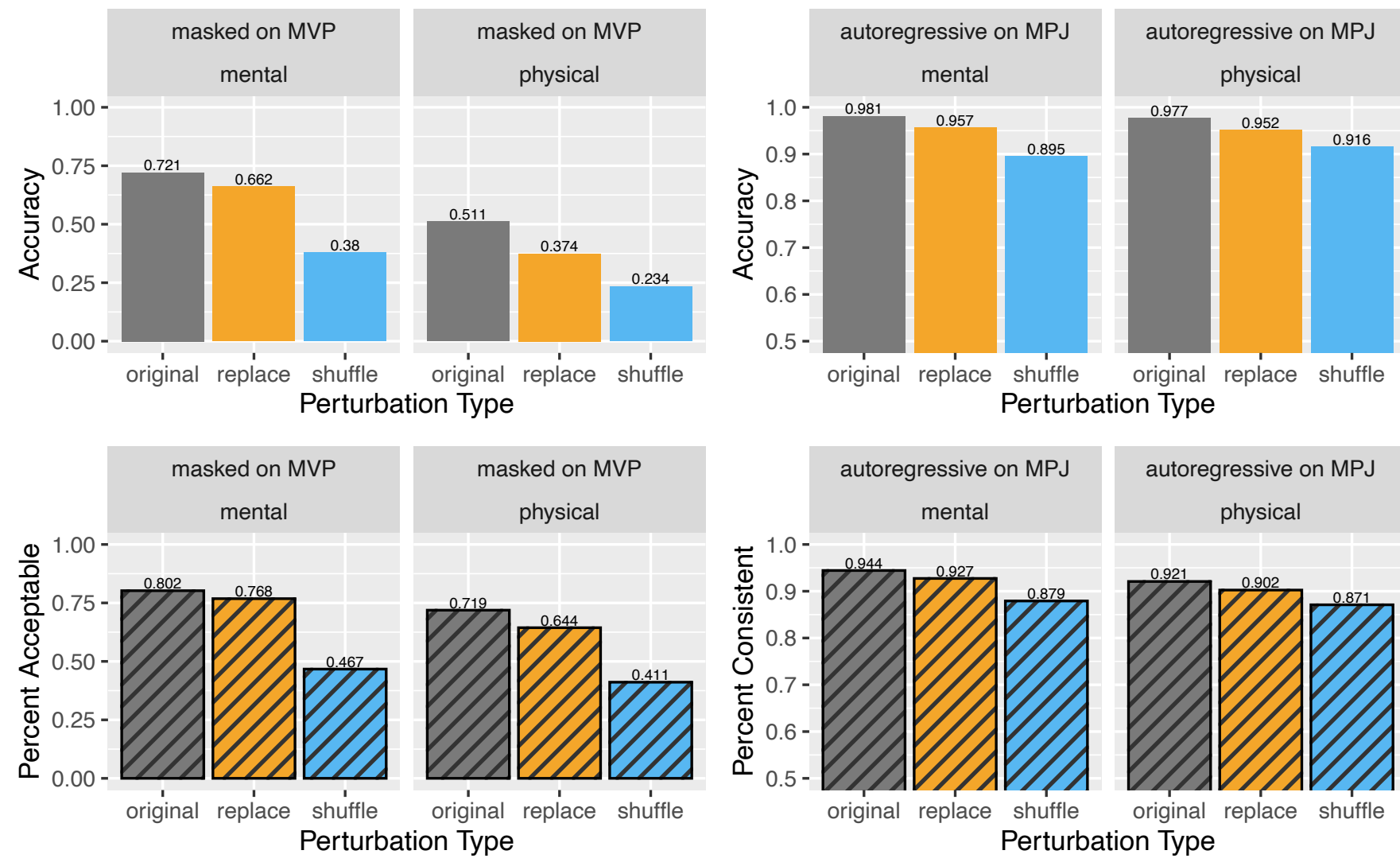

Figure B.2. Mental vs. physical distinction under the accuracy-based metric (top row) and the alternative metrics (bottom row).

Mental vs. Physical verbs We examine whether the mental vs. physical distinction we observed under the accuracy-based metric holds under the two LLM-based metrics. Results are shown in Figure B.2. We ran the same statistical model as in the accuracy-based metric, but predicting acceptability/consistency instead. We found the same statistical patterns as in the accuracy-based metric. For the masked model, the REPLACE.WORD x physical interaction is negative, and the SHUFFLE.ORDER x physical interaction is positive (Table B.2). For the autoregressive model, both interactions are positive, but the magnitude of the interaction is larger in the SHUFFLE.ORDER x physical case (Table B.3). Altogether, the results under the LLM-based metrics validate our previous findings: there is an asymmetry between mental and physical verbs in terms of their dependence on co-occurrence information vs. structural information.

Parameter	Log-Odds	SE	95% CI	p
(Intercept)	1.34	0.16	[1.02, 1.66]	$< .001$
perturbation [replace]	-0.20	0.02	[-0.25, -0.16]	$< .001$
perturbation [shuffle]	-1.55	0.02	[-1.60, -1.51]	$< .001$
verb type [physical]	-0.40	0.21	[-0.81, 0.00]	0.051
perturbation [replace] × verb type [physical]	-0.16	0.04	[-0.23, -0.09]	$< .001$
perturbation [shuffle] × verb type [physical]	0.17	0.03	[0.10, 0.24]	$< .001$

Table B.2. Logistic mixed-effects model predicting masked model acceptability with verb type and perturbation type.

Impact of Shuffling Units We examine whether our results regarding the impact of shuffling units hold under the LLM-based metrics. Results are shown in Figure B.3. Again, the pattern here is qualitatively consistent with the accuracy-based metric.

Parameter	Log-Odds	SE	95% CI	p
(Intercept)	3.02	0.10	[2.83, 3.21]	$< .001$
perturbation [replace]	-0.29	0.02	[-0.33, -0.26]	$< .001$
perturbation [shuffle]	-0.88	0.02	[-0.91, -0.84]	$< .001$
verb type [physical]	-0.69	0.22	[-1.11, -0.27]	0.001
perturbation [replace] × verb type [physical]	0.06	0.03	[0.01, 0.11]	0.021
perturbation [shuffle] × verb type [physical]	0.32	0.02	[0.28, 0.37]	$< .001$

Table B.3. Logistic mixed-effects model predicting autoregressive model consistency with verb type and perturbation type.

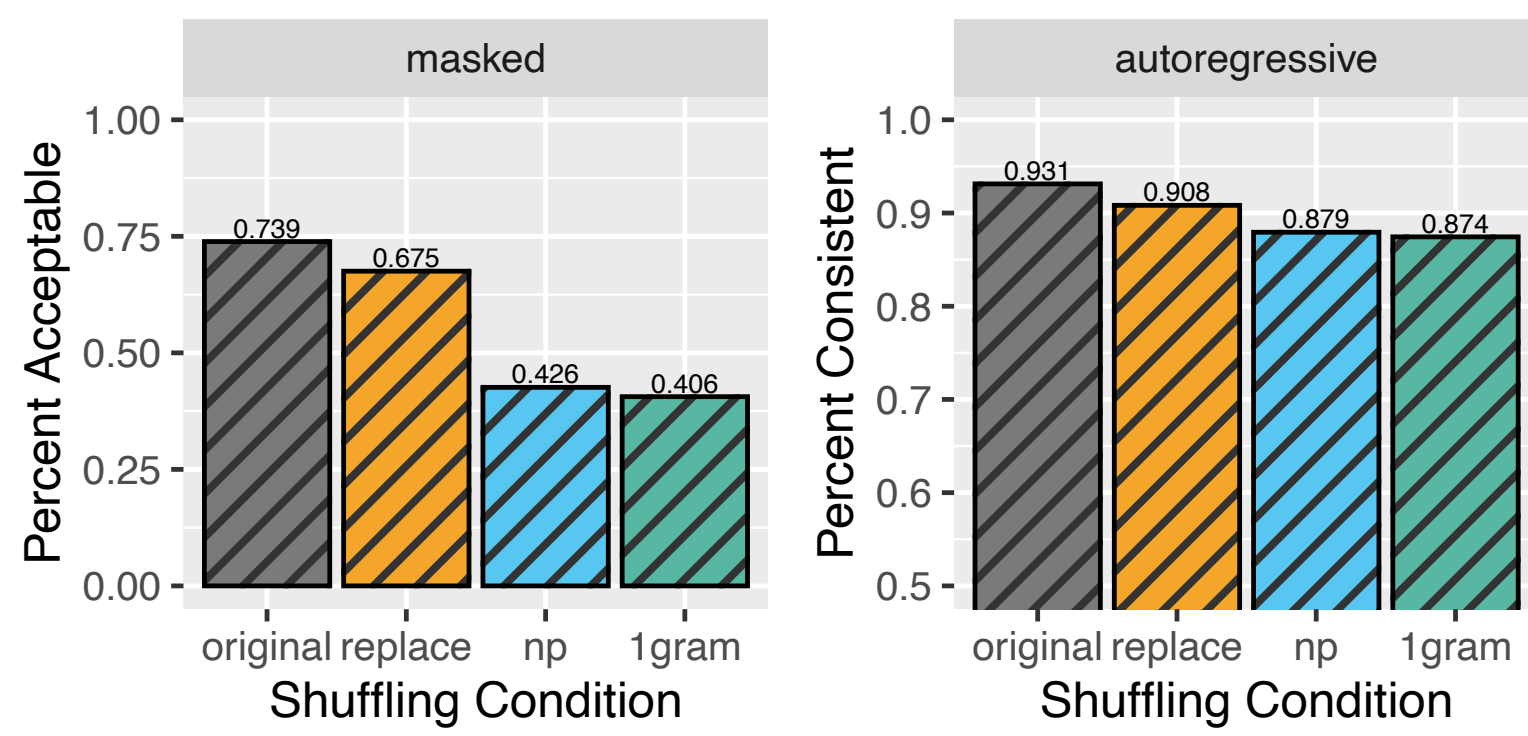

Figure B.3. Effect of shuffling unit evaluated under the LLM-based metrics.

C: SENTENCE RANDOMIZER ALGORITHM

We followed Sinha et al. (2021)'s SentenceRandomizer algorithm to create the SHUFFLE.ORDER perturbation. The pseudocode for this algorithm is listed in Algorithm 1.

D: TRAINING TRAJECTORIES

Training trajectories for our masked models and autoregressive models are shown in Figure D.1 and Figure D.2, respectively.

E: EMBEDDING ANALYSIS

We provide visualizations of noun vs. verb embeddings after PCA for both the masked model (Figure E.1) and the autoregressive model (Figure E.2). We do not find evidence that models under the SHUFFLE.ORDER condition failed to learn reliable noun-verb distinctions.

Algorithm 1 SentenceRandomizer

procedure $\mathcal{F}(S, t, n)$ ▷ Randomize a sentence S with seed t and n grams n
W = tokenize the words in S
Set the seed to t
if $n > 1$ **then**
while True **do**
K = Sample all possible starting points from $[0, |W| - n]$
Ignore the starting points in K which overlap with conjoined tokens ▷ Conjoined tokens consists of joined unigrams
if $|K| \geq 1$ **then**
Sample one position $p \in K$
g = Extract the n-gram $W[p : p + n]$
Delete $W[p + 1 : p + n]$
$W[p]$ = Convert g to a conjoined token
else
Break from While loop
end if
end while
end if
while True **do**
$\hat{W}$ = randomly shuffle tokens in W
$r = \sum(\hat{W}[i] = W[i])$ ▷ Count number of positions where the token remains in its original position
if $r = 0$ **then** Break out of While loop
end if
end while
$\hat{S}$ = join the tokens in $\hat{W}$
Return $\hat{S}$
end procedure

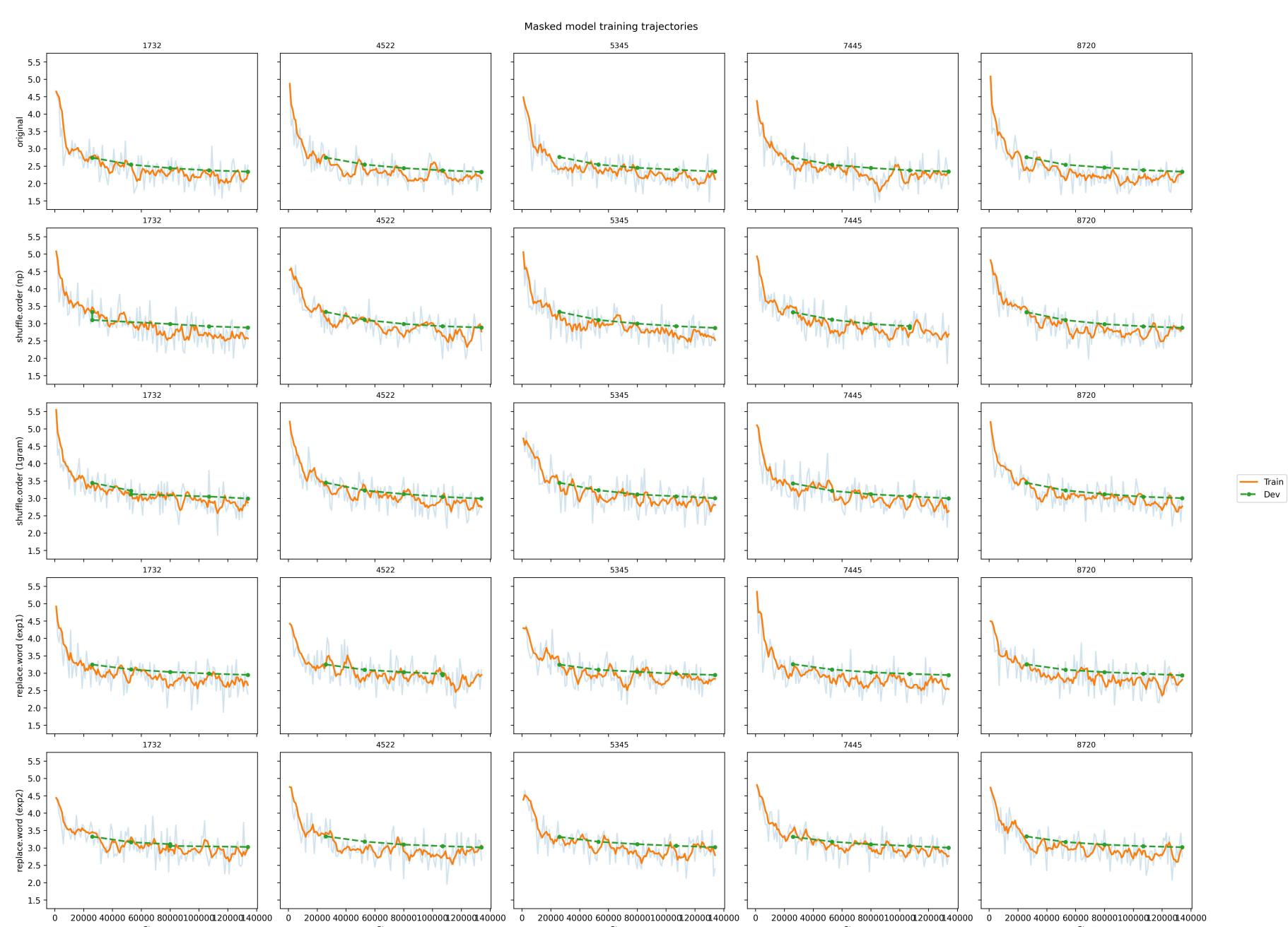

Figure D.1. Training and validation loss curves for masked models by random seed and perturbation type. Training loss was recorded every 1000 training steps, while dev loss was evaluated per epoch.

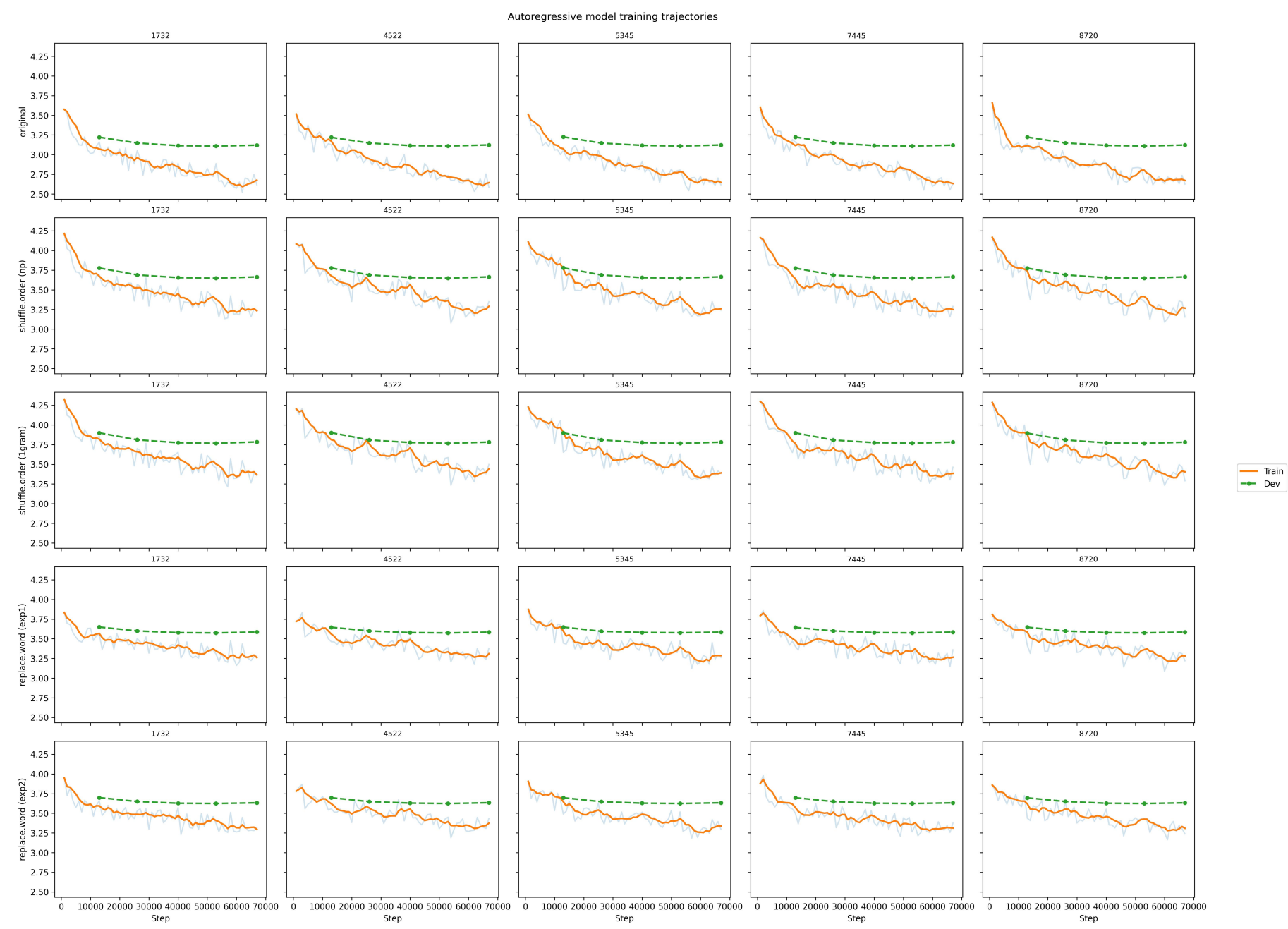

Figure D.2. Training and validation loss curves for autoregressive models by random seed and perturbation type.

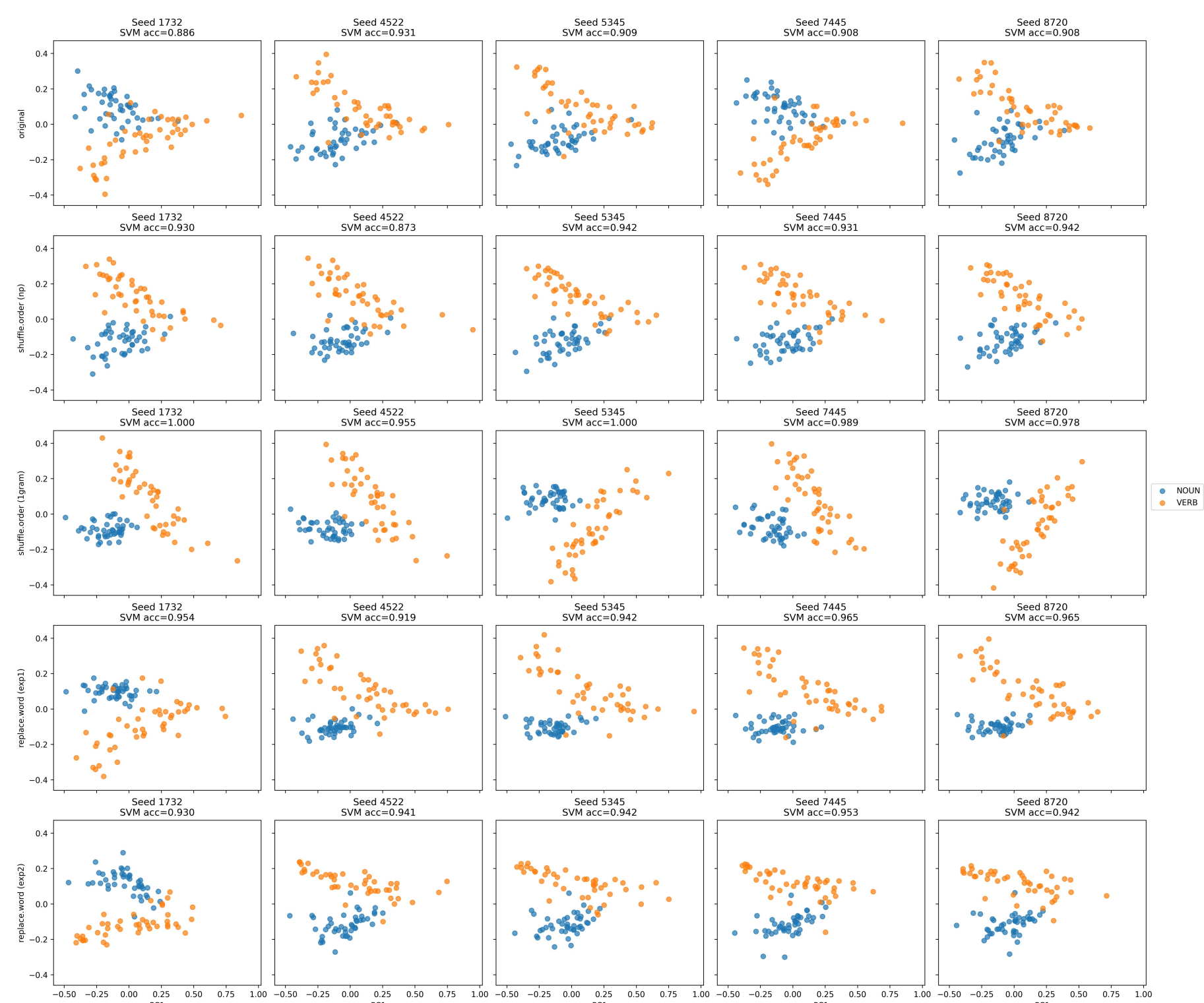

Figure E.1. Visualization of noun vs. verb embeddings after PCA for the masked model. Rows correspond to different perturbations and columns to different random seeds.

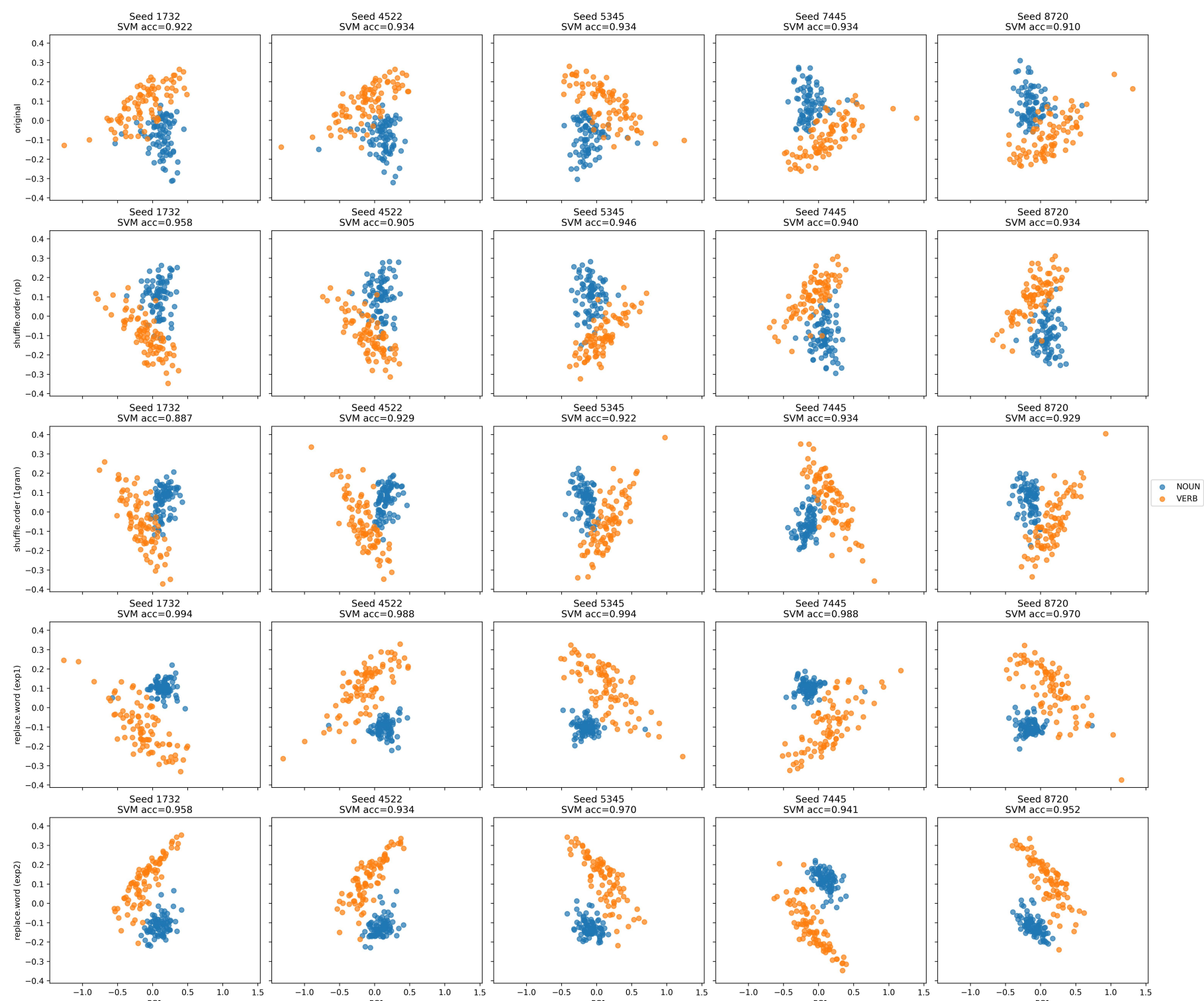

Figure E.2. Visualization of noun vs. verb embeddings after PCA for the autoregressive model. Rows correspond to different perturbations and columns to different random seeds.
